# Prognostic Risk Factors in Randomized Clinical Trials of Face-to-Face and Internet-Based Psychotherapy for Depression: A Systematic Review and Meta-Regression Analysis

**DOI:** 10.1001/jamapsychiatry.2023.3861

**Published:** 2023-10-11

**Authors:** Mariia Merzhvynska, Markus Wolf, Tobias Krieger, Thomas Berger, Thomas Munder, Birgit Watzke

**Affiliations:** 1 Department of Psychology, University of Zurich, Zurich, Switzerland; 2 Department of Psychology, University of Bern, Bern, Switzerland

## Abstract

**Importance:**

Variables such as severe symptoms, comorbidity, and sociodemographic characteristics (eg, low educational attainment or unemployment) are associated with a poorer prognosis in adults treated for depressive symptoms. The exclusion of patients with a poor prognosis from RCTs is negatively associated with the generalizability of research findings.

**Objective:**

To compare the prognostic risk factors (PRFs) in patient samples of RCTs of face-to-face therapy (FTF) and internet-based therapy (IBT) for depression.

**Data Sources:**

PsycINFO, Cochrane CENTRAL, and reference lists of published meta-analyses were searched from January 1, 2000, to December 31, 2021.

**Study Selection:**

RCTs that compared FTF (individual or group therapy) and IBT (guided or self-guided interventions) against a control (waitlist or treatment as usual) in adults with symptoms of depression were included.

**Data Extraction and Synthesis:**

Data were extracted by 2 independent observers. The Cochrane revised risk-of-bias tool was used to assess the risk of bias. The study was preregistered with OSF Registries and followed the Preferred Reporting Items for Systematic Reviews and Meta-analyses (PRISMA) reporting guideline.

**Main Outcomes and Measures:**

The primary outcome was the standardized mean difference (Hedges *g* effect size) in depressive symptoms at treatment termination (assessed with standard patient self-report questionnaires), with a positive standardized mean difference indicating larger improvements in the intervention compared with those in the control group. Meta-regression analyses were adjusted for the type of control group. Three preregistered and 2 exploratory sensitivity analyses were conducted. A prognostic risk index (PROG) was created that calculated the sum of 12 predefined individual indicators, with scores ranging from 0 to 12 and higher scores indicating that a sample comprised patients with poorer prognoses.

**Results:**

This systematic review and meta-regression analysis identified 105 eligible RCTs that comprised 18 363 patients. In total, 48 studies (46%) examined FTF, and 57 studies (54%) examined IBT. The PROG was significantly higher in the RCTs of FTF than in the RCTs of IBT (FTF: mean [SD], 3.55 [1.75]; median [IQR], 3.5 [2.0-4.5]; IBT: mean [SD], 2.27 [1.66]; median [IQR], 2.0 [1.0-3.5]; *z* = −3.68, *P* < .001; Hedges *g* = 0.75; 95% CI, 0.36-1.15). A random-effects meta-regression analysis found no association of the PROG with the effect size. Sensitivity analyses with outliers excluded and accounting for risk of bias or small-study effects yielded mixed results on the association between the PROG and effect size.

**Conclusions and Relevance:**

The findings of this systematic review and meta-regression analysis suggest that samples of RCTs of FTF vs IBT differ with regard to PRFs. These findings have implications for the generalizability of the current evidence on IBT for depression. More RCTs of internet-based interventions with clinically representative samples are needed, and the reporting of PRFs must be improved.

## Key Points

**Question:**

Do samples of randomized clinical trials (RCTs) of face-to-face therapy (FTF) and internet-based therapy (IBT) for depression differ with regard to the prognostic risk factors (ie, prognosis) of the included patients?

**Findings:**

In this systematic review and meta-regression analysis of 105 RCTs comprising 18 363 participants, the prevalence of patients with poor prognosis was higher in RCTs of FTF than in the RCTs of IBT. The quality of reporting of prognostic risk factors was not optimal.

**Meaning:**

These results suggest that indirect comparisons of FTF and IBT may be problematic because, in terms of reporting prognostic risk factors, samples of RCTs may not be drawn from the same clinical population.

## Teaser

This systematic review and meta-regression analysis compares the prognostic risk factors in patient samples reported in 105 randomized clinical trials of face-to-face and internet-based psychotherapy for treating depression.

## Introduction

Psychotherapy for depression can be offered in different settings and treatment modalities. Traditionally, treatment is delivered face to face, but internet-based therapy (IBT) has gained popularity and the number of randomized clinical trials (RCTs) of IBT has grown during the past 2 decades.^1^ Recent direct and indirect comparisons^2,3^ suggest that therapist-guided IBT can be as beneficial as face-to-face therapy (FTF).

To draw valid inferences about different treatments for routine care, RCTs should include patients representative of the clinical population. However, while the rationale of RCTs maximizes the internal validity, the external validity or generalizability (ie, whether the effects can be generalized to the patient population in clinical practice) is an issue, with up to 80% of individuals with depression being excluded from depression trials due to their failure to meet inclusion criteria.^4,5^ This includes patients with unfavorable socioeconomic characteristics and complex clinical presentations^6^ (ie, factors known to be associated with a poorer prognosis).^7,8^ Yet it remains unclear whether the empirical distributions of prognostic risk factors (PRFs) in samples in FTF and IBT trials differ or not.^9,10^

This study was preregistered with OSF Registries.^11^ We aimed to compare the samples of RCTs of FTF and IBT for depression with regard to PRFs and explore their association with outcome.

### Methods

This systematic review and meta-regression analysis followed the Preferred Reporting Items for Systematic Reviews and Meta-Analyses (PRISMA) reporting guideline (eTable 1 in Supplement 1) and used the Cochrane revised risk-of-bias tool to assess risk of within-study bias (eMethods in Supplement 1).^12,13^ We included RCTs that investigated the efficacy of FTF (individual or group) or IBT (guided or self-guided) in adults with acute depressive symptoms compared with either a treatment as usual or waiting list control group. PsycINFO, Cochrane CENTRAL, and the reference lists of published meta-analyses were searched from January 1, 2000, to December 31, 2021 (eTable 2 in Supplement 1).

A prognostic risk index (PROG) was created to quantify in each trial the extent to which participants with PRFs were enrolled. The PROG comprised 12 predefined PRFs (eg, diagnosis of depression, and lower income) from the literature (eMethods 2 and eTable 8 in Supplement 1). Three factors were coded dichotomously indicating the presence (1) or absence (0) of participants with a given prognostic factor, and 9 were coded on a scale including 1, 0.5, and 0. If information was not assessed, not reported, or unclear, the factor was coded as absent (0). The PROG was equal to the sum of the factors ranging from 0 to 12, with higher values representing a sample that comprised patients with a more unfavorable prognosis. The interrater reliability (intraclass coefficient using 2-way random-effects and absolute agreement) for PROG was 0.87 (95% CI, 0.80-0.91; *F* = 7.87; *P* < .001).

We used the Mann-Whitney *U* and Kruskal-Wallis tests to assess PROG differences between trials investigating FTF and IBT and their submodalities (individual FTF, group FTF, guided IBT, and self-guided IBT). Fisher exact and χ^2^ tests were used to assess differences in the frequencies of single prognostic factors. Meta-regression analyses estimated the association between PROG and outcome. The primary outcome was the standardized mean difference (Hedges *g* effect size) in depressive symptoms at treatment termination, with a positive standardized mean difference indicating larger improvements in the intervention compared with those in the control group. Meta-regression analyses were adjusted for type of control group.^14^ Three preregistered and 2 exploratory sensitivity analyses were conducted. The threshold for statistical significance was 2-tailed (*P* = .05).

## Results

Our literature search identified 105 eligible trials (eFigure 1 and eTable 3 in Supplement 1), randomizing 18 363 participants. The FTF was examined in 48 trials (46%) comprising 4073 participants and IBT in 57 trials (54%) comprising 14 290 participants.

The overall mean (SD) PROG was 2.86 (1.81); the median (IQR) PROG was 3.0 (1.5-4.0). The PROG was higher in studies of FTF than in IBT (FTF: mean [SD], 3.55 [1.75]; median [IQR], 3.5 [2.0-4.5]; IBT: mean [SD], 2.27 [1.66]; median [IQR], 2.0 [1.0-3.5]; *z* = −3.68, *P* < .001; Hedges *g* = 0.75 [95% CI, 0.36-1.15]). The PROG varied among the 4 submodalities (Figure), with individual FTF scoring the highest and self-guided IBT scoring lowest (χ^2^_3_ = 20.6; *P* < .001).

**Figure.**
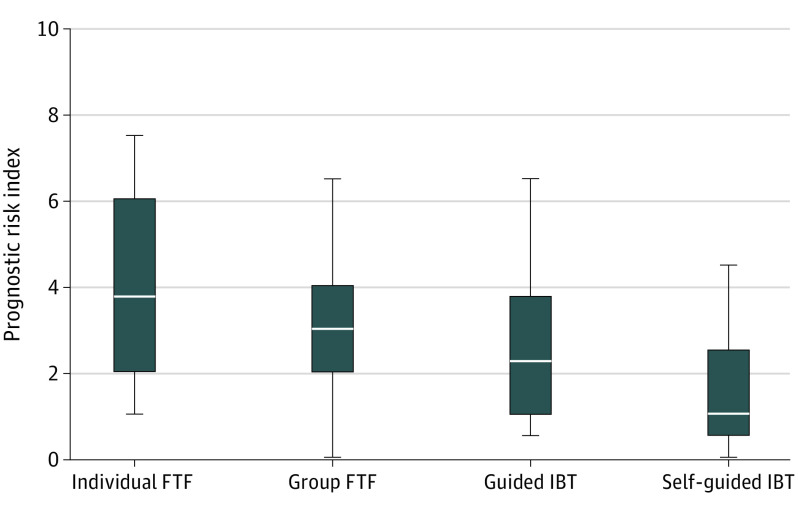
Prognostic Risk Index (PROG) as a Function of 4 Treatment Submodalities in Samples of Randomized Clinical Trials on Face-to-Face Therapy (FTF) and Internet-Based Therapy (IBT) for Depression The box plot shows the empirical distribution of the PROG in patient samples. The white horizontal lines indicate the median; the boxes indicate the first and third quartiles; and the whiskers, the minimum and maximum values.

A clinical diagnosis of a depressive disorder (51% of the trials) and chronic or recurrent depression (34%) were the individual PRFs most frequently accounted for in the trials (eTable 4 in Supplement 1). Comorbid personality disorders (FTF 8.3% vs IBT 0%; *P* = .04), a clinical diagnosis of depression (FTF 72.9% vs IBT 31.6%; *P* < .001), severe depressive symptoms (FTF 35.4% vs IBT 3.5%; *P* < .001), and low education (FTF 39.6% vs IBT 15.8%; *P* = .008) were significantly more prevalent in FTF than in IBT (eTable 5 in Supplement 1).

The amount of data not assessed or not reported for each of the 12 PRFs ranged from 9% (diagnosis of depression) to 88% (comorbid personality disorder) (eTable 6 in Supplement 1). Not-reported PRF data were more frequent in IBT (mean [SD], 6.91 [1.97]) than in FTF (mean [SD], 5.67 [1.84]; *z* = 2.97; *P* = .003).

A random-effects meta-regression analysis suggested that PROG was not associated with effect size (Table). Between-study heterogeneity of effect sizes was large (*I^2^* = 93%). After exclusion of 4 outliers (Hedges *g *> 2.00), PROG was associated with outcome (B = 0.05 [95% CI, 0.02-0.09]; SE = 0.02; *P* = .006). Two sensitivity analyses, which accounted for risk of bias (low risk vs other) and small-study size (N < 100) indicated no association between PROG and outcome. Exploratory sensitivity analyses with outliers removed indicated that the association between PROG and outcome was not significant after controlling for treatment modality (Table).

**Table.** Random-Effects Meta-Regression Analysis of Prognostic Risk Factors in Trials of Therapy for Depression vs Control^a^

Model	Studies, No.	Variable	Coefficient (95% CI)	SE	*P* value	*I*^2^, %
All trials	105	Intercept	0.52 (0.30 to 0.74)	0.11	<.001	93
		Type of control	−0.10 (−0.22 to 0.01)	0.06	.08	
		PROG^b^	0.05 (−0.01 to 0.12)	0.03	.11	
Preregistered sensitivity analyses						
Outliers excluded	101	Intercept	0.41 (0.28 to 0.53)	0.06	<.001	76
		Type of control	−0.14 (−0.20 to −0.07)	0.04	<.001	
		PROG	0.05 (0.01 to 0.08)	0.02	.006	
Trials with n ≥100	49	Intercept	0.49 (0.22 to 0.76)	0.14	<.001	96
		Type of control	−0.04 (−0.20 to 0.12)	0.08	.66	
		PROG	0.01 (−0.08 to 0.08)	0.04	.88	
Adjusted for risk of bias	105	Intercept	0.42 (0.18 to 0.66)	0.12	.001	92
		Type of control	−0.08 (−0.21 to 0.03)	0.06	.14	
		Risk of bias	−0.14 (−0.31 to 0.01)	0.08	.07	
		PROG	0.05 (−0.01 to 0.12)	0.03	.10	
Additional sensitivity analyses						
Outliers excluded, adjusted for 2 modalities	101	Intercept	0.38 (0.26 to 0.51)	0.06	<.001	74
		Type of control	−0.14 (−0.21 to −0.08)	0.03	<.001	
		PROG	0.03 (−0.01 to 0.06)	0.02	.12	
		Modality	0.22 (0.07 to 0.36)	0.08	.003	
Outliers excluded, adjusted for 4 submodalities^c^	93	Intercept	0.34 (0.21 to 0.48)	0.07	<.001	72
		Type of control	−0.14 (−0.21 to −0.07)	0.04	<.001	
		PROG	0.01 (−0.02 to 0.06)	0.02	.50	
		Guided IBT	0.17 (−0.002 to 0.34)	0.09	.05	
		Individual FTF	0.34 (0.12 to 0.55)	0.11	.003	
		Group FTF	0.30 (0.10 to 0.50)	0.10	.003	

Abbreviations: FTF, face-to-face psychotherapy; IBT, internet-based therapy; PROG, prognostic risk index.

^a^ Coefficient refers to the meta-regression coefficient; restricted maximum likelihood was used.

^b^ The value indicates that a 1-point increase in PROG was associated with a 0.05-point increase in the effect size (Hedges *g*) that compared the intervention against the control group.

^c^ Self-guided IBT served as the reference group.

## Discussion

This systematic review and meta-regression analysis found that samples of RCTs of FTF more often included patients with PRFs than RCTs of IBT. An explanation might be that these trials tend to include self-selected samples from the community or online sources, as opposed to FTF trials, which more frequently enroll patients from clinical settings.^15^

Our results regarding the association of PROG and outcome are inconclusive, which might be due to the study design, its sample size, or the composition of the PROG. The reporting quality of PRFs was poor in most trials, particularly in IBT trials. This renders the translation of trial results difficult in clinical practice, where most patients present with complex conditions. Based on these findings, comparisons across different depression treatments are difficult because trial samples are possibly not drawn from the same clinical population. To improve, reporting guidance is needed about which PRFs are deemed relevant, and adherence to guidelines should be encouraged by grant providers and scientific journals.

### Limitations

This study has limitations. In total, 90 of 105 trials were judged at a risk of bias or some concern. We assigned equal weights to all indicators, but research has yet to determine whether they are of equal relevance. If no information about a PRF was available, we treated this factor as absent, but we cannot be sure if this was actually the case. Our sample size was probably too small for subgroup analyses to reliably address the associations between PROG and outcome.

## Conclusions

This systematic review and meta-regression analysis suggests that trials of IBT and FTF for depression may differ with regard to PRFs in their samples; thus, inferences about the benefits of depression treatments delivered by FTF vs IBT are difficult to draw. Future RCTs should recruit clinically representative samples, and the reporting of PRFs needs to be improved.

## Notes

Supplement 1. **eMethods 1.** Risk of Bias Assessment **eMethods 2.** Rationale and Development of the Prognostic Risk Index (PROG) **eFigure 1.** PRISMA Flow Diagram **eTable 1.** PRISMA 2009 Checklist **eTable 2.** Search Algorithm **eTable 3.** Included Trials Contrasting Face-to-Face and Internet-based Psychotherapy for Depression with Control Groups **eTable 4.** Frequency of 12 Individual Prognostic Risk Factors in RCTs of Four Therapy Submodalities **eTable 5.** Distributions of 12 Individual Prognostic Risk Factors in RCTs of Internet and Face-to-Face Therapy **eTable 6.** Distribution of 12 Individual Prognostic Risk Factor Ratings Including Not Reported Data **eTable 7.** Frequency of Not Reported Data on Each Prognostic Risk Factor in Four Therapy Submodalities **eTable 8.** Extract From the Codebook with Definitions of Prognostic Risk Factors **eReferences** ^1^**.** Trials Included in the Systematic Review Contrasting Face-to-Face and Internet-Based Therapy for Depression **eReferences** ^2^**.** Literature Informing the Development of a Prognostic Risk Index in Depression

Supplement 2.
Data Sharing Statement
