# Supplementary material for: Prognostic Risk Factors in Randomized Clinical Trials of Face-to-Face and Internet-Based Psychotherapy for Depression: A Systematic Review and Meta-Regression Analysis
Source: JAMA Psychiatry. 2023 Oct 11;81(1):97–100. doi: 10.1001/jamapsychiatry.2023.3861 (PMC10568439; doi:10.1001/jamapsychiatry.2023.3861)
Supplement: Supplement 1. — eMethods 1. Risk of Bias Assessment eMethods 2. Rationale and Development of the Prognostic Risk Index (PROG) eFigure 1. PRISMA Flow Diagram eTable 1. PRISMA 2009 Checklist eTable 2. Search Algorithm eTable 3. Included Trials Contrasting Face-to-Face and Internet-based Psychotherapy for Depression with Control Groups eTable 4. Frequency of 12 Individual Prognostic Risk Factors in RCTs of Four Therapy Submodalities eTable 5. Distributions of 12 Individual Prognostic Risk Factors in RCTs of Internet and Face-to-Face Therapy eTable 6. Distribution of 12 Individual Prognostic Risk Factor Ratings Including Not Reported Data eTable 7. Frequency of Not Reported Data on Each Prognostic Risk Factor in Four Therapy Submodalities eTable 8. Extract From the Codebook with Definitions of Prognostic Risk Factors eReferences 1. Trials Included in the Systematic Review Contrasting Face-to-Face and Internet-Based Therapy for Depression eReferences 2. Literature Informing the Development of a Prognostic Risk Index in Depression [file jamapsychiatry-e233861-s001.pdf]

Supplementary Online Content

Merzhvynska M, Wolf M, Krieger T, Berger T, Munder T, Watzke B. Prognostic risk factors in randomized clinical trials of face-to-face and internet-based psychotherapy for depression. *JAMA Psychiatry.* Published online October 11, 2023. doi:10.1001/jamapsychiatry.2023.3861

eMethods 1: Risk of Bias Assessment

eMethods 2: Rationale and Development of the Prognostic Risk Index (PROG)

eFigure 1: PRISMA Flow Diagram

eTable 1: PRISMA 2009 Checklist

eTable 2: Search Algorithm

eTable 3: Included Trials Contrasting Face-to-Face and Internet-Based Therapy for Depression with Control Groups

eTable 4: Frequency of 12 Individual Prognostic Risk Factors in RCTs of Four Therapy Submodalities

eTable 5: Distributions of 12 Individual Prognostic Risk Factors in RCTs of Internet-Based and Face-to-Face Therapy

eTable 6: Distribution of 12 individual Prognostic Risk Factor Ratings Including Not Reported Data

eTable 7: Frequency of Not Reported Data on Each Prognostic Risk Factor in Four Therapy Submodalities

eTable 8: Extract from the Codebook with Definitions of Prognostic Risk Factors

eReferences 1: Trials Included in the Systematic Review Contrasting Face-to-Face and Internet-Based Therapy for Depression

eReferences 2: Literature Informing the Development of a Prognostic Risk Index in Depression

© 2023 Merzhvynska M et al. *JAMA Psychiatry.*

eMethod 1. Risk of Bias Assessment

Cochrane's revised risk of bias tool was used to assess risk of within-study bias from five domains (randomization process, deviations from intended interventions, missing outcome data, outcome assessment, and selective reporting). Trials fulfilling all five domains were considered low risk; all other trials were considered to be at some concern or high risk of bias. Risk-of-bias results for each of the included studies can be found in eTable 3.

© 2023 Merzhvynska M et al. *JAMA Psychiatry.*

eMethod 2. Rationale and development of the Prognostic Risk Index (PROG)

Rationale of the Prognostic Risk Index:

The primary aim of the development of the prognostic risk index for the current study was to enable the comparison of the generalizability of samples included in internet and face-to-face psychotherapy trials based on an evidence-based, yet parsimonious approach. To the best of our knowledge, no previous research has developed a single index that can be applied for the purposes of our study, i.e., to compare the generalizability of trial samples across a broad range of psychotherapy approaches and treatment modalities. While there is a growing interest in clinical prediction models for prognosis of treatment outcome, models which have been tested thus far are fragmentary with regard to the selected variables and have often used within-treatment process information (e.g. Kessler, 2018; for the list of references see additional online material on OSF https://osf.io/yspr6/files/osfstorage). Most importantly, these models share a different focus, with most prognostic models being specified and optimized for outcome prediction or individual treatment selection regarding a defined set of treatment alternatives, rather than aiming at a broader prognosis of depression (e.g., PAI, Cohen & DeRubeis, 2018; LRI, Delgadillo et al., 2020). Given the evidence on the clinical relevance of combining single prognostic factors (e.g., Buckman et al., 2021), we decided to develop an evidence-based index, which summarizes well-recognized prognostic risk factors that should be reported when conducting a psychotherapy outcome study or RCT. Thus, a Prognostic Risk Index was created to cover clinically relevant patient characteristics, i.e., characteristics that have shown to be negatively associated with depression outcome, and to reflect the current state of research.

Development of the Prognostic Risk Index:

The development of the Prognostic Risk Index was based on two steps: 1) A scoping review (a focused, non-systematic review of the current literature aiming at identifying the most relevant factors); 2) an expert consensus process.

Ad 1. As a first step, a scoping review was conducted to provide an overview of existing prognostic factors in depression and treatment outcome research (see eTable 8, and Table “Overview of the literature informing the development of a Prognostic Risk Index in depression ” on OSF https://osf.io/yspr6/files/osfstorage). Two criteria were decisive for the consideration and inclusion of a given prognostic risk factor in the current study: (1) The prognostic risk factor had to be valid and clinically well-recognized to be associated with depression outcome. We considered a broad range of literature and evidence stemming from depression research or based on findings from clinical and social epidemiology (described in, e.g., authoritative reports on social determinants of well-being, (observational) prospective studies or systematic reviews); (2) The prognostic risk factor had to be relevant for psychotherapy research: This means we focused on factors that are typically reported in RCTs and psychotherapy outcome research, and have thus proven feasible for inclusion our study which aimed to cover the most common psychotherapy treatment modalities. PsycINFO and Pubmed as well as the reference lists of included studies were searched, with a focus on recent literature. In step 1, we identified a broad range of factors that have been used for various empirical purposes including the ones described above (e.g., prediction, prognosis, treatment selection, differential indication, effect moderators, etc.). The results of our unsystematic review revealed a larger group of

© 2023 Merzhvynska M et al. *JAMA Psychiatry.*

variables that are derived from clinical-psychological and psychotherapy research, covering factors such as depressive symptom severity, having received a full diagnosis, chronicity of the disorder, comorbid mental disorders, personality or substance abuse disorder. Another large group of variables was based on clinical- and social-epidemiological research and covered important person characteristics such as older age, gender, low education, low income, unstable employment status, or belonging to an ethnic minority (see eReferences 2 below and additional online material “Overview of the literature informing the development of a Prognostic Risk Index in depression” on OSF https://osf.io/yspr6/files/osfstorage).

Ad 2. As a second step, the individual prognostic risk factors were discussed in a series of consensus meetings. A list of prognostic risk factors was compiled and the final set of variables to be included in this study was determined through an expert consensus process led by the authors (BW, MW, TB, TK, TM). Furthermore, while a growing body of research is focusing on the identification of patient factors relevant for individual treatment selection, there is no consensus in the literature about the relative weight or importance each factor should be given (e.g., Buckman et al., 2021; see additional online table “Overview of the literature informing the development of a Prognostic Risk Index in depression” on OSF https://osf.io/yspr6/files/osfstorage). Therefore, based on the current research evidence, the consensus group decided not to attribute different weights to individual prognostic risk factors. Furthermore, a methodological challenge within the context of depression and psychotherapy is that “a variable can function as a prognostic predictor in one context and as a prescriptive predictor in another” (Cohen & DeRubeis, 2018; p. 211), so often no clear distinction has been made between baseline characteristics that are associated with poor outcomes in terms of natural history of depression (prognostic variables), versus baseline variables that have an impact on the effects of an intervention (i.e., outcome moderators, effect modifiers, prescriptive variables) (e.g., Kessler et al., 2017). Whereas this distinction is well grounded on a conceptual level, from a psychotherapeutic perspective this distinction, thus, might appear not as straight forward or even artificial, as the core function of psychotherapy (and an important competency of psychotherapists) is to adjust treatments to patients, i.e., leverage treatment based on the patient’s individual needs and characteristics (e.g., therapist responsiveness) which is “is ubiquitous and creates serious problems for a ballistic, cause–effect understanding of how psychotherapy works“ (Kraemer & Stiles, 2015; p. 1). Because the primary aim of our study was to assess the generalizability of trials on internet-based versus face-to-face therapy, and based on the fact that of these variables have been used for either purpose, we decided to combine them referring to these variables as “prognostic risk factors”. The fact that the index is a mix of both factors needs to be considered when interpreting the results of analyses that address the associations of the index with therapy outcome from comparative research. We finally considered 12 single “prognostic risk factors” clinically relevant to be included in our index (for details see eTable 8): Depressive symptom severity; full clinical diagnosis of a depressive disorder; chronicity of the depression; comorbid mental disorder; comorbid personality disorder; comorbid substance use disorder; no partner; lower education; no occupation; belonging to an ethnic minority; older age. Three factors were coded dichotomously indicating the presence (1) or absence (0) of participants with a given prognostic factor, and nine factors were coded on a 3-point scale (1, 0.5, 0), which allowed to assess a factor’s partial presence (see eTable 8). In case information was not assessed, missing, not

© 2023 Merzhvynska M et al. *JAMA Psychiatry.*

reported, or unclear in a given study, the factor was coded as absent (0). PROG was equal to the sum of the factors ranging from zero to 12 with higher values representing a sample that consisted of patients with more unfavorable prognosis.

References

Buckman JEJ, Saunders R, Cohen ZD, et al. The contribution of depressive 'disorder characteristics' to determinations of prognosis for adults with depression. An individual patient data meta-analysis. *Psychol Med.* 2021;51(7):1068–1081. doi:10.1017/S0033291721001367.

Cohen ZD, DeRubeis RJ. Treatment Selection in Depression. *Annu Rev Clin Psychol.* 2018;14:209–236. doi:10.1146/annurev-clinpsy-050817-084746.

Delgadillo J, Appleby S, Booth S, et al. The Leeds Risk Index. Field-Test of a Stratified Psychological Treatment Selection Algorithm. *Psychother Psychosom.* 2020;89(3):189–190. doi:10.1159/000505193.

Kessler RC, van Loo HM, Wardenaar KJ, et al. Using patient self-reports to study heterogeneity of treatment effects in major depressive disorder. *Epidemiol Psychiatr Sci.* 2017;26(1):22–36. doi:10.1017/S2045796016000020.

Kessler RC. The potential of predictive analytics to provide clinical decision support in depression treatment planning. *Curr Opin Psychiatry.* 2018;31(1):32–39. doi:10.1097/YCO.0000000000000377.

Kraemer U, Stiles WB. The responsiveness problem in psychotherapy. A review of proposed solutions. *Clinical Psychology: Science and Practice.* 2015;22(3):277–295. doi:10.1111/cpsp.12107.

© 2023 Merzhvynska M et al. *JAMA Psychiatry.*

eFigure 1. PRISMA flow diagram

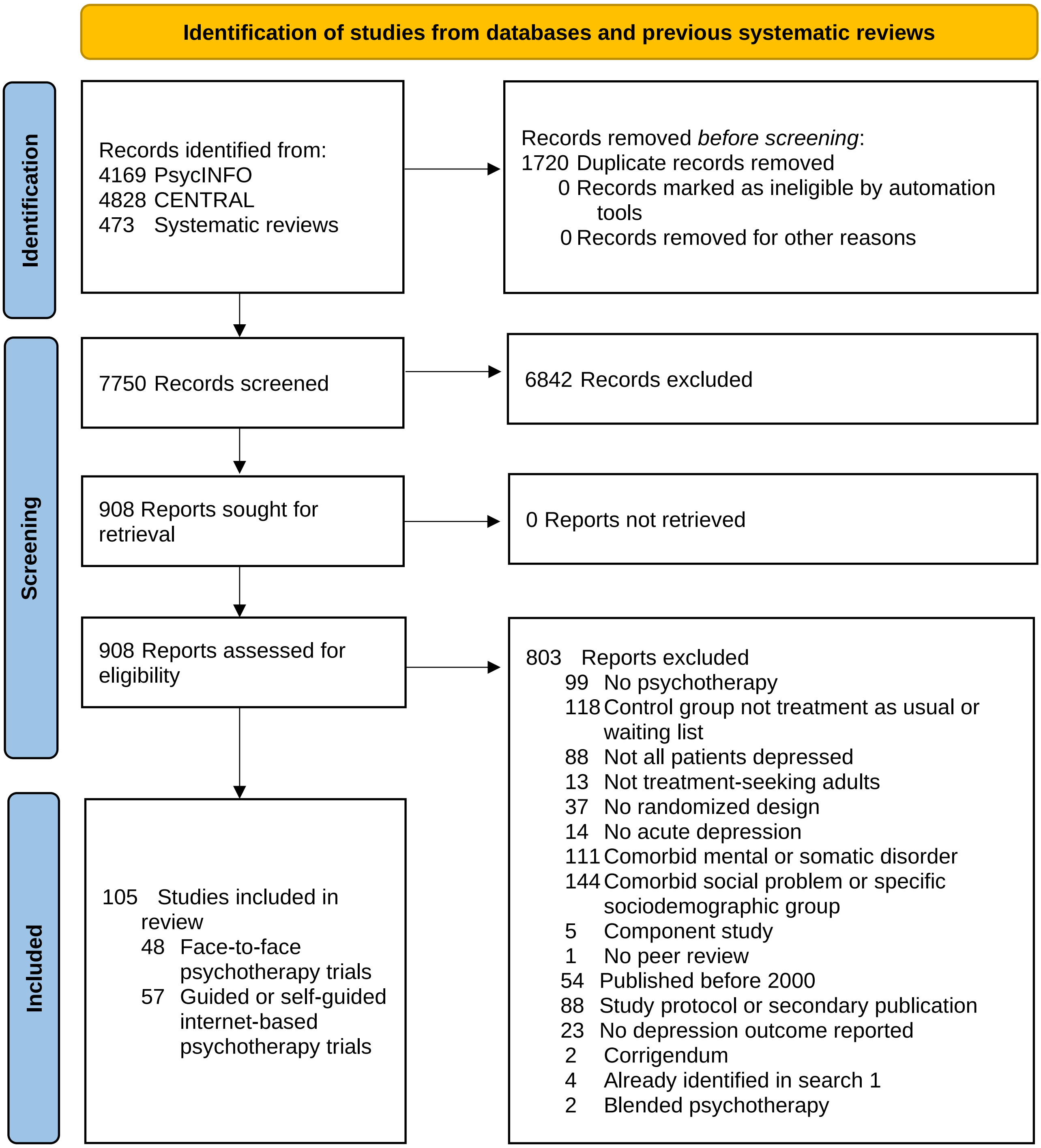

© 2023 Merzhvynska M et al. *JAMA Psychiatry.*

eTable 1. PRISMA 2009 Checklist

Section/topic	#	Checklist item	Reported on page #
**TITLE**			
Title	1	Identify the report as a systematic review, meta-analysis, or both.	1, title page
**ABSTRACT**			
Structured summary	2	Provide a structured summary including, as applicable: background; objectives; data sources; study eligibility criteria, participants, and interventions; study appraisal and synthesis methods; results; limitations; conclusions and implications of key findings; systematic review registration number.	1
**INTRODUCTION**			
Rationale	3	Describe the rationale for the review in the context of what is already known.	2
Objectives	4	Provide an explicit statement of questions being addressed with reference to participants, interventions, comparisons, outcomes, and study design (PICOS).	2
**METHODS**			
Protocol and registration	5	Indicate if a review protocol exists, if and where it can be accessed (e.g., Web address), and, if available, provide registration information including registration number.	2
Eligibility criteria	6	Specify study characteristics (e.g., PICOS, length of follow-up) and report characteristics (e.g., years considered, language, publication status) used as criteria for eligibility, giving rationale.	2, study information at OSF
Information sources	7	Describe all information sources (e.g., databases with dates of coverage, contact with study authors to identify additional studies) in the search and date last searched.	2, eTable 2
Search	8	Present full electronic search strategy for at least one database, including any limits used, such that it could be repeated.	4, eTable 2
Study selection	9	State the process for selecting studies (i.e., screening, eligibility, included in systematic review, and, if applicable, included in the meta-analysis).	2, eFigure 1
Data collection process	10	Describe method of data extraction from reports (e.g., piloted forms, independently, in duplicate) and any processes for obtaining and confirming data from investigators.	2, study information at OSF
Data items	11	List and define all variables for which data were sought (e.g., PICOS, funding sources) and any assumptions and simplifications made.	2, eTable 8 and OSF coding sheet
Risk of bias in individual studies	12	Describe methods used for assessing risk of bias of individual studies (including specification of whether this was done at the study or outcome level), and how this information is to be used in any data synthesis.	2, eMethod1
Summary measures	13	State the principal summary measures (e.g., risk ratio, difference in means).	2

© 2023 Merzhvynska M et al. *JAMA Psychiatry.*

Section/topic	#	Checklist item	Reported on page #
Synthesis of results	14	Describe the methods of handling data and combining results of studies, if done, including measures of consistency (e.g., I^2) for each meta-analysis.	2
Risk of bias across studies	15	Specify any assessment of risk of bias that may affect the cumulative evidence (e.g., publication bias, selective reporting within studies).	eMethod1
Additional analyses	16	Describe methods of additional analyses (e.g., sensitivity or subgroup analyses, meta-regression), if done, indicating which were pre-specified.	2, study information at OSF
**RESULTS**			
Study selection	17	Give numbers of studies screened, assessed for eligibility, and included in the review, with reasons for exclusions at each stage, ideally with a flow diagram.	eFigure 1
Study characteristics	18	For each study, present characteristics for which data were extracted (e.g., study size, PICOS, follow-up period) and provide the citations.	eTable 3; OSF coding sheet
Risk of bias within studies	19	Present data on risk of bias of each study and, if available, any outcome level assessment (see item 12).	eTable 3
Results of individual studies	20	For all outcomes considered (benefits or harms), present, for each study: (a) simple summary data for each intervention group (b) effect estimates and confidence intervals, ideally with a forest plot.	2-3, eTable 3, Table in Manuscript
Synthesis of results	21	Present results of each meta-analysis done, including confidence intervals and measures of consistency.	Table in Manuscript
Risk of bias across studies	22	Present results of any assessment of risk of bias across studies (see Item 15).	Table in Manuscript
Additional analysis	23	Give results of additional analyses, if done (e.g., sensitivity or subgroup analyses, meta-regression [see Item 16]).	Table in Manuscript
**DISCUSSION**			
Summary of evidence	24	Summarize the main findings including the strength of evidence for each main outcome; consider their relevance to key groups (e.g., healthcare providers, users, and policy makers).	3
Limitations	25	Discuss limitations at study and outcome level (e.g., risk of bias), and at review-level (e.g., incomplete retrieval of identified research, reporting bias).	3
Conclusions	26	Provide a general interpretation of the results in the context of other evidence, and implications for future research.	3
**FUNDING**			

© 2023 Merzhvynska M et al. *JAMA Psychiatry.*

Funding	27	Describe sources of funding for the systematic review and other support (e.g., supply of data); role of funders for the systematic review.	No funding

From: Moher D, Liberati A, Tetzlaff J, Altman DG; PRISMA Group. Preferred reporting items for systematic reviews and meta-analyses: the PRISMA statement. BMJ. 2009;339:b2535. doi:10.1136/bmj.b2535

© 2023 Merzhvynska M et al. *JAMA Psychiatry.*

eTable 2. Search algorithm

**Identification of RCTs in CENTRAL and PsycINFO**
Randomized controlled trial (applied in All Text):<br>random* or ((control* or compar* or clin*) N3 (trial* or studi* or study))<br><br>Psychotherapy (applied in Title, Abstract, and Keywords):<br>(Psychotherap* or ((acceptance or attachment or behavio#r* or analytic* or brief or cognitive or dynamic or psychodynamic or emotion* or feminist or sensiti?ation or existen* or exposure or gestalt or humanistic or hypno* or integr* or short-term* or shortterm* or long-term* or longterm* or interpersonal* or inter-personal* or mindfulness* or multimodal or narrative or positive or psychol* or rational* or solution* or eclectic* or experiential* or expressive* or insight* or supportive* or schema*) N5 (treatment* or therapy or therapies or psychotherapy or intervention*))) or (((web or digital or Internet or online or computer or app* or application* or smartphone or smart-phone) N5 (therapy or therapies or psychotherapy or intervention* or treatment*)) or ((guided or unguided or assisted or therapist*) N5 self*) or e-therapy or icbt)<br><br>Depression (applied in Title and Keywords):<br>depression or depressive or dysthymi*
**Identification of systematic reviews in PsycINFO[a]**
Systematic review methodology (applied in Title):<br>meta-analy* or (systematic review)<br><br>Psychotherapy and Depression sections were identical to the randomized trials search described above.

Abbreviation: CENTRAL, Cochrane Central Register of Controlled Trials.
[a] Search terms given in EBSCO syntax for PsycINFO.

© 2023 Merzhvynska M et al. *JAMA Psychiatry.*

eTable 3. Included trials contrasting face-to-face and internet-based therapy for depression with control groups

Study	Intervention			Type of control	Risk of bias[b]	Outcome measure	PROG
	Treatment	Modality	No. of participants				
Addington et al. 2019	Positive emotion skills intervention	Self-guided IBT	15	WL	some concern or high	PHQ-8	2.5
						CES-D	
Andersson et al. 2005	iCBT	Guided IBT	60	WL	some concern or high	BDI-I	1.5
						MADRS	
Anuwatgasem et al., 2020	mindfulness and self-compassion-based group therapy	Group FTF	52	TAU	some concern or high	MADRS	4.0
						HADS-D	
Arroll et al., 2022	FACT	Individual FTF	57	WL	some concern or high	PHQ	2.0
Barnhofer et al. 2009	MBCT	Group FTF	16	TAU	some concern or high	BDI-II	3.0
Beevers et al. 2017	Deprexis	Self-guided IBT	91	TAU/WL	low	QIDS-SR	2.5
						HRSD	
Berger et al. 2011	Deprexis (with support)	Guided IBT	26	WL	some concern or high	BDI-II	6.0
	Deprexis (without support)	Self-guided IBT					
Berking et al. 2019	Affect regulation training	Group FTF	72	WL	some concern or high	BDI and HRSD combined	4.0
Bohlmeijer et al. 2011	ACT	Group FTF	44	WL	some concern or high	CES-D	3.0
Bolier et al. 2013	Psyfit	Self-guided IBT	141	WL	some concern or high	CES-D	1.0

Bucker et al. 2019	Mood	Self-guided IBT	63	TAU	some concern or high	BDI-II	0.5
						PHQ-9	
Buntrock et al. 2015	GET.ON mood enhancer	Guided IBT	204	TAU	some concern or high	CES-D	0.5
Carlbring et al. 2013	BA + ACT	Guided IBT	40	WL	some concern or high	MADRS	2.0
						BDI-II	
Carr et al. 2017	Say yes to life	Group FTF	42	TAU	some concern or high	BDI-II	4.0
						HRSD	
						MADRS	
Carta et al. 2012	Counselling	Individual FTF	42	TAU	some concern or high	BDI-I	4.5
Castonguay et al. 2004	Integrative cognitive therapy	Individual FTF	14	WL	some concern or high	BDI-I	2.0
						HRSD	
Chan et al. 2012	CBT	Group FTF	25	WL	some concern or high	HRSD	3.0
						BDI-II	
Chiang et al. 2015	GCBT	Group FTF	40	TAU	some concern or high	BDI-II	3.5
						HRSD	
Cladder-Micus et al. 2018	MBCT	Group FTF	57	TAU	some concern or high	IDS	4.5
Clarke et al. 2002	Depression skills program	Self-guided IBT	116	TAU	some concern or high	CES-D	0.5
Clarke et al. 2005	Depression skills program (with postcard reminders)	Self-guided IBT	79	TAU	some concern or high	CES-D	0.5
	Depression skills program (with telephone reminders)	Self-guided IBT					

© 2023 Merzhvynska M et al. *JAMA Psychiatry.*

Dahne et al. 2019	Moodivate	Self-guided IBT	9	TAU	some concern or high	BDI-II	3.0
	Moodkit	Self-guided IBT					
De Graaf et al. 2009	iCBT	Self-guided IBT	103	TAU	low	BDI-II	1.0
Ebert et al. 2018	iCBT	Guided IBT	102	TAU/WL	some concern or high	QIDS-SR	0.5
						HRSD	
						CES-D	
Embling et al. 2002	CBT	Group FTF	19	WL	some concern or high	BDI-II	2.0
Everitt et al., 2021	ImproveYourMood	Self-guided IBT	235	WL	some concern or high	PHQ-9	1.5
	ImproveYourMood+	Self-guided IBT					
Farrer et al. 2011	iCBT (with telephone tracking)	Guided IBT	35	TAU	some concern or high	CES-D	2.0
	iCBT (without telephone tracking)	Self-guided IBT					
Fonagy et al. 2015	LTPP	Individual FTF	62	TAU	some concern or high	HRSD	6.5
						BDI-II	
Forand et al. 2018	iCBT	Guided IBT	30	WL	some concern or high	PHQ-9	5.0
						HRSD	
Fuhr et al. 2018	Deprexis	Self-guided IBT	14	WL	some concern or high	PHQ-9	0
Geraedts et al. 2014	Happy@work	Guided IBT	115	TAU	some concern or high	CES-D	1.0
Gibbons et al. 2012	SET	Individual FTF	19	TAU	some concern or high	HRSD	6.0
Gilbody et al. 2015	Beating the Blues	Self-guided IBT	239	TAU	low	PHQ-9	2.5

© 2023 Merzhvynska M et al. *JAMA Psychiatry.*

	MoodGym	Self-guided IBT					
Gräfe et al., 2020	Deprexis	Self-guided IBT	3805	TAU	some concern or high	PHQ-9	3.0
Hagen et al. 2017	MCT	Individual FTF	19	WL	some concern or high	BDI-I	5.0
Hallgren et al. 2015	iCBT	Guided IBT	312	TAU	some concern or high	MADRS	6.5
Harley et al. 2008	DBT	Group FTF	11	WL	some concern or high	HRSD	2.0
						BDI-I	
Hemanny et al., 2020	TBCT	Individual FTF	76	TAU	some concern or high	HRSD	5.0
	BA					BDI	
Holas et al., 2020	MBCT	Group FTF	81	WL	some concern or high	CES-D	3.5
Jelinek et al., 2020	internet-based behavioral activation (iBA)	Self-guided IBT	61	TAU	some concern or high	PHQ-9	1.5
Johansson et al. 2019	iCBT	Guided IBT	27	WL	some concern or high	MADRS	5.5
						HADS-D	
Jurinec et al., 2020	Coping with depression course	Group FTF	84	WL	some concern or high	BDI-II	0.5
Kenter et al. 2016	PST	Guided IBT	133	WL	some concern or high	CES-D	4.0
King et al. 2000	CBT	Individual FTF	67	TAU	some concern or high	BDI-I	5.5
	non-directive counselling	Individual FTF					
Kivi et al. 2014	iCBT	Guided IBT	47	TAU	some concern or high	BDI-II	1.0
						MADRS	
Kleiboer et al. 2015	PST (without guidance)	Self-guided IBT	106	WL	low	CES-D	2.0

© 2023 Merzhvynska M et al. *JAMA Psychiatry.*

	PST (with guidance on request)	Guided IBT				PHQ-9	
	PST (with weekly guidance)	Guided IBT					
Klein et al. 2016	Deprexis	Guided IBT	504	TAU	low	PHQ-9	3.5
						HRSD	
						IDS	
Krämer et al., 2021	WBI	Guided IBT	136	TAU	low	CES-D	1.5
						PHQ-9	
Lambert et al. 2018	BA + physical activity	Self-guided IBT	30	WL	some concern or high	PHQ-8	1.0
Lappalainen et al. 2015	ACT	Guided IBT	20	WL	some concern or high	BDI-II	3.5
Lee et al., 2021	BA	Group FTF	64	TAU	some concern or high	HRSD	3.0
						CES-D	
Lemma et al. 2013	DIT (with therapist facilitation)	Guided IBT	8	WL	some concern or high	PHQ-9	0.5
	DIT (without therapist facilitation)	Self-guided IBT					
Lynch et al. 2020	DBT	Individual FTF	88	TAU	some concern or high	HRSD	7.0
						PHQ-9	
Löbner et al. 2018	iCBT	Self-guided IBT	327	TAU	low	BDI-II	3.0
						PHQ-9	
Lüdtke et al. 2018	Be good to yourself	Self-guided IBT	45	WL	some concern or high	PHQ-9	0
MacLean et al., 2020	Coach-guided web-based therapy The Journal	Guided IBT	93	TAU	low	PHQ-9	1.0
MacPherson et al. 2013	Counselling	Individual FTF	151	TAU	low	PHQ-9	3.5

© 2023 Merzhvynska M et al. *JAMA Psychiatry.*

Mahmoodi et al., 2020	CBT focused on perfectionism	Individual FTF	75	WL	some concern or high	BDI-II	1.0
	Unified Protocol for transdiagnostic treatment of emotional disorders (UP)	Individual FTF					
Maina et al. 2005	PDT	Individual FTF	10	WL	some concern or high	HRSD	2.0
	Supportive therapy	Individual FTF					
Meyer et al. 2009	Deprexis	Self-guided IBT	76	TAU/WL	some concern or high	BDI-I	3.5
Meyer et al. 2015	Deprexis	Self-guided IBT	85	TAU/WL	some concern or high	PHQ-9	1.0
Michalak et al. 2015	MBCT	Group FTF	35	TAU	some concern or high	HRSD	4.5
	CBASP	Group FTF				BDI-II	
Mira et al. 2017	Smiling is fun (automated support)	Self-guided IBT	44	WL	some concern or high	BDI-II	1.0
	Smiling is fun (human support)	Guided IBT				ODSIS	
Mohr et al. 2013	Mood manager (with telephone support)	Guided IBT	33	WL	some concern or high	PHQ-9	4.5
	Mood manager (without telephone support)	Self-guided IBT					
Montero-Marin et al. 2016	Smiling is Fun (therapist-guided)	Guided IBT	102	TAU	some concern or high	BDI-II	3.5
	Smiling is Fun (self-guided)	Self-guided IBT					
Moritz et al. 2012	Deprexis	Self-guided IBT	105	WL	some concern or high	BDI-I	0.5
Mukhtar et al. 2011	GCBT	Group FTF	55	TAU	some concern or high	BDI-I	2.0
Naeem et al. 2015	CBT	Individual FTF	68	TAU	some concern or high	HADS-D	3.5

© 2023 Merzhvynska M et al. *JAMA Psychiatry.*

Niedermoser et al., 2020	W-IPT	Group FTF	28	TAU	some concern or high	HRSD	3.0
						BDI-II	
Nyström et al. 2017	BA based on Lewisohn	Guided IBT	53	WL	some concern or high	PHQ-9	3.5
	BA based on Martell	Guided IBT					
Omidi et al. 2013	MBCT	Group FTF	30	TAU	some concern or high	BSI-D	2.0
	CBT	Group FTF					
Perini et al. 2009	Sadness program	Guided IBT	19	WL	some concern or high	PHQ-9	3.0
						BDI-II	
Pots et al. 2014	MBCT	Group FTF	75	WL	some concern or high	CES-D	1.5
Pots et al. 2016	ACT	Guided IBT	87	WL	some concern or high	CES-D	1.5
Richards et al. 2015	iCBT	Guided IBT	129	WL	low	BDI-II	4.0
Rief et al. 2018	CBASP	Individual FTF	42	WL	some concern or high	BDI-II	2.5
	CBT (with physical exercise)	Individual FTF					
	CBT (with mindfulness tasks)	Individual FTF					
Roepke et al. 2015	SuperBetter (version with CBT and PPT)	Self-guided IBT	93	WL	some concern or high	CES-D	1.0
	SuperBetter (general version)	Self-guided IBT					
Rohricht et al. 2013	Body psychotherapy	Group FTF	15	WL	some concern or high	HRSD	4.0
Rosso et al. 2017	iCBT	Self-guided IBT	40	WL	low	HRSD	4.5
						PHQ-9	
Ruwaard et al. 2009	iCBT	Guided IBT	18	WL	some concern or high	BDI-I	3.0

© 2023 Merzhvynska M et al. *JAMA Psychiatry.*

						SCL-90-D	
Schramm et al., 2020	W-IPT	Group FTF	28	TAU	some concern or high	HRSD	3.0
						BDI-II	
Schuling et al., 2020	MBCL	Group FTF	122	TAU	some concern or high	BDI-II	3.0
Schure et al. 2019	iCBT	Self-guided IBT	162	WL	some concern or high	PHQ-9	1.0
Schuster et al. 2017	PPT	Group FTF	24	WL	some concern or high	CES-D	0
Segal et al., 2020	MMB	Guided IBT	460	TAU	low	PHQ-9	2.5
Seligman et al. 2006	PPT	Individual FTF	15	TAU	some concern or high	ZSRS	3.5
Sinniah et al. 2017	CBT	Individual FTF	36	TAU	some concern or high	BDI-I	4.0
						DASS-D	
Smith et al. 2017	iCBT	Self-guided IBT	68	WL	low	PHQ-9	4.5
Stiles-Shields et al. 2019	BT	Guided IBT	10	WL	some concern or high	PHQ-9	0.5
	CT	Guided IBT					
Strauss et al. 2012	CT	Group FTF	14	TAU	some concern or high	BDI-II	6.5
Sugg et al. 2018	Morita therapy	Individual FTF	34	TAU	some concern or high	PHQ-9	3.5
Szumska et al. 2021	Mindfulness-based cognitive therapy	Group FTF	20	WL	some concern or high	HADS-D	4.0
						CES-D	
Titov et al. 2010	iCBT (technician-assisted)	Self-guided IBT	45	WL	some concern or high	PHQ-9	4.0
	iCBT (clinician-assisted)	Guided IBT				BDI-II	

© 2023 Merzhvynska M et al. *JAMA Psychiatry.*

Tong et al. 2020	GCBT	Group FTF	45	WL	some concern or high	HRSD	2.5
Town et al. 2017	ISTDP	Individual FTF	30	TAU	some concern or high	HRSD	6.5
						PHQ-9	
Tulbure et al. 2018	iCBT (conventional)	Guided IBT	26	WL	some concern or high	BDI-II	1.5
	iCBT (religious)	Guided IBT				IDS	
Vernmark et al. 2010	iCBT (individualized email therapy)	Guided IBT	29	WL	some concern or high	BDI-I	5.5
	iCBT (guided self-help)	Guided IBT				MADRS	
Warmerdam et al. 2008	iCBT	Guided IBT	87	WL	low	CES-D	1.0
	PST	Guided IBT					
Wiersma et al. 2014	CBASP	Individual FTF	72	TAU	some concern or high	IDS	7.0
Wiles et al. 2013	CBT	Individual FTF	234	TAU	low	BDI-I	7.5
						PHQ-9	
Williams AD et al. 2013	iCBT	Self-guided IBT	31	WL	some concern or high	BDI-II	2.5
						PHQ-9	
Wong et al. 2008	CBT	Group FTF	48	WL	some concern or high	BDI-I	3.0
Wright et al. 2005	CT	Individual FTF	15	WL	some concern or high	HRSD	1.5
						BDI-II	
Yeung et al. 2018	MoodGym	Self-guided IBT	38	TAU	some concern or high	CES-D	0.5
Zu et al. 2014	CBT	Individual FTF	30	TAU	some concern or high	HRSD	2.0

© 2023 Merzhvynska M et al. *JAMA Psychiatry.*

QIDS-SR

Abbreviations: ACT, acceptance and commitment therapy; BA, behavioural activation; BDI-I, Beck Depression Inventory; BDI-II, Beck Depression Inventory revised; BSI-D, Brief Symptom Inventory Depression subscale; BT, behavioural therapy; CBASP, cognitive-behavioural analysis system of psychotherapy; CBT, cognitive-behaviour therapy; CES-D, Center for Epidemiological Studies Depression scale; CT, cognitive therapy; DASS-D, Depression Anxiety Stress Scale Depression subscale; DBT, dialectical behaviour therapy; DIT, dynamic-interpersonal therapy; FTF, face-to-face psychotherapy; FACT, Focused acceptance and commitment therapy; GCBT, cognitive-behaviour group therapy; HADS-D, Hospital Depression and Anxiety Scale - depression subscale; HRSD, Hamilton Rating Scale for Depression; iCBT, internet-based cognitive-behavioural therapy; IDS, Inventory for Depressive Symptomatology; IBT, internet-based therapy; ISTDP, intensive short-term dynamic psychotherapy; LTPP, long-term psychoanalytic psychotherapy; MADRS, Montgomery-Åsberg Depression Rating Scale; MBCL, Mindfulness-based compassionate living; MBCT, mindfulness-based cognitive therapy; MCT, metacognitive therapy; MMB, Mindful Mood Balance Treatment; ODSIS, Overall Depression Severity and Impairment Scale; PDT, psychodynamic therapy; PHQ-8, PHQ-9, Patient Health Questionnaire; PPT, positive psychotherapy; PROG, index of prognostic risk factors; PST, problem-solving therapy; QIDS-SR, Quick Inventory of Depressive Symptomatology; SCL-90-D, Symptom Check List-90 Depression subscale; SET, supportive-expressive therapy; TAU, Treatment as Usual; TBCT, Trial-based cognitive therapy; WBI, Web-based intervention; W-IPT, Workplace-related Interpersonal Psychotherapy; WL, Waiting List; ZSRS, Zung Self-Rating Scale.

[a] Trials with lower intensity fulfilled 0 intensity indicator, trials with higher intensity fulfilled ≥ 1 intensity indicator(s).

[b] Trials with some concern or high risk of bias have some concern or high risk of bias on ≥ 1 risk of bias domain; trials with low risk of bias have low risk of bias on 5 risk of bias domains.

© 2023 Merzhvynska M et al. *JAMA Psychiatry.*

eTable 4. Frequency of 12 individual prognostic risk factors in RCTs of four therapy submodalities[a]

		Therapy submodality				
	Total	Ind FTF	Group FTF	Guided IBT	Self-guided IBT	P Value
Prognostic risk factor	No. (%)	No. (%)	No. (%)	No. (%)	No. (%)	
Symptom severity	19 (18.1)	9 (40.9)	8 (30.8)	0 (0)	2 (8.0)	**.001**
Diagnosis	53 (50.5)	16 (72.7)	19 (73.1)	11 (45.8)	3 (12.0)	**<.001**
Chronic or recurrent depression	35 (33.3)	9 (40.9)	12 (46.2)	8 (33.3)	4 (16.0)	.12
Comorbid mental disorders	18 (17.0)	9 (40.9)	3 (11.5)	2 (8.3)	2 (8.0)	**.006**
Comorbid personality disorders	4 (3.8)	4 (18.2)	0 (0)	0 (0)	0 (0)	**.003**
Comorbid substance use disorder	7 (6.7)	3 (13.6)	1 (3.8)	2 (8.3)	0 (0)	.24
No college	28 (26.7)	8 (36.4)	11 (42.3)	4 (16.7)	4 (16.0)	.08
Low income	11 (10.5)	4 (18.2)	2 (7.7)	3 (12.5)	1 (4.0)	.41
No occupation	16 (15.2)	5 (22.7)	6 (23.1)	3 (12.5)	1 (4.0)	.19
Ethnic minority	10 (9.5)	5 (22.7)	0 (0)	1 (4.2)	3 (12.0)	**.04**
Single/no partner	9 (8.5)	1 (4.5)	4 (15.4)	0 (0)	2 (8.0)	.19
Age > 65 years	28 (26.7)	6 (27.3)	3 (11.5)	11 (45.8)	5 (20.0)	**.04**

Abbreviations: Ind FTF, individual face-to-face therapy (k = 22); Group FTF, group face-to-face therapy (k = 26); Guided IBT, guided internet-based therapy (k = 24); Self-guided IBT, self-guided internet-based therapy (k = 25).

[a] This table presents prognostic risk factors indicating the presence (= 1) of participants with a given prognostic risk factor in RCTs in the total sample (k = 97) and four therapy submodalities.

© 2023 Merzhvynska M et al. *JAMA Psychiatry.*

eTable 5. Distributions of 12 individual prognostic risk factors in RCTs of internet-based and face-to-face therapy[a, b]

	Therapy modality						
	IBT (k = 57)			FTF (k = 48)			
Prognostic risk factor	Present No. (%)	Partly present No. (%)	Absent No. (%)	Present No. (%)	Partly present No. (%)	Absent No. (%)	P Value
Symptom severity	2 (3.5)	34 (59.6)	21 (36.8)	17 (35.4)	10 (20.8)	21 (43.8)	**<.001**
Diagnosis	18 (31.6)	6 (10.5)	33 (57.9)	35 (72.9)	8 (16.7)	5 (10.4)	**<.001**
Chronic or recurrent depression	14 (24.6)	2 (3.5)	41 (71.9)	21 (43.8)	2 (4.2)	25 (52.1)	
Comorbid mental disorder	6 (10.5)	4 (7.0)	47 (82.5)	12 (25.0)	7 (14.6)	29 (60.4)	.07
Comorbid personality disorder	0 (0)	0 (0)	57 (100.0)	4 (8.3)	2 (4.2)	42 (87.5)	**.04**
Comorbid substance use disorder	3 (5.3)	n/a	54 (94.7)	4 (8.3)	n/a	44 (91.7)	.70
No college	9 (15.8)	9 (15.8)	39 (68.4)	19 (39.6)	8 (16.7)	21 (43.8)	**.008**
Low income	5 (8.8)	n/a	52 (91.2)	6 (12.5)	n/a	42 (87.5)	.54
No occupation	5 (8.8)	7 (12.3)	45 (78.9)	11 (22.9)	4 (8.3)	33 (68.8)	.058
Ethnic minority	5 (8.8)	3 (5.3)	49 (86.0)	5 (10.4)	1 (2.1)	42 (87.5)	>.99
Single/no partner	4 (7.0)	14 (24.6)	39 (68.4)	5 (10.4)	3 (6.3)	40 (83.3)	.73
Age > 65 years	19 (33.3)	n/a	38 (66.7)	9 (18.8)	n/a	39 (81.3)	.12

Abbreviations: n/a, not applicable (i.e., "partly present" was not coded for this variable). IBT, internet-based therapy (k = 57), FTF, face-to-face therapy (k = 48).

[a] This table indicates the presence (1), partial presence (0.5) or absence (0, incl. not reported) of participants with a given prognostic risk factor in modalities of internet-based and face-to-face therapy.

[b] A table with distributions for each prognostic risk factor with and without not reported data can be found at OSF https://osf.io/yspr6/files/osfstorage.

© 2023 Merzhvynska M et al. *JAMA Psychiatry.*

eTable 6. Distribution of 12 individual prognostic risk factor ratings including not reported data[a]

	Rating of prognostic risk factor			
Prognostic risk factor	Present<br>No. (%)	Partly present<br>No. (%)	Not present<br>No. (%)	Not reported<br>No. (%)
Symptom severity	19 (18.1)	44 (41.9)	7 (6.7)	35 (33.3)
Diagnosis	53 (50.5)	14 (13.3)	29 (27.6)	9 (8.6)
Chronic or recurrent depression	35 (33.3)	4 (3.8)	7 (6.7)	59 (56.2)
Comorbid mental disorders	18 (17.1)	11 (10.5)	11 (10.5)	65 (61.9)
Comorbid personality disorders	4 (3.8)	2 (1.9)	7 (6.7)	92 (87.6)
Comorbid substance use disorder	7 (6.7)	n/a	47 (44.8)	51 (48.6)
No college	28 (26.7)	17 (16.2)	33(31.4)	27 (25.7)
Low income	11 (10.5)	n/a	4 (3.8)	90 (85.7)
No occupation	16 (15.2)	11 (10.5)	13 (12.4)	65 (61.9)
Ethnic minority	10 (9.5)	4 (3.8)	15 (14.3)	76 (72.4)
Single/no partner	9 (8.6)	17 (16.2)	18 (17.1)	61 (58.1)
Age > 65 years	28 (26.7)	n/a	41 (39)	36 (34.3)

Abbreviation: n/a, not applicable (i.e., "partly present" was not coded for this variable).
[a.] This table indicates the presence (1), partial presence (0.5) or absence (0) of participants with a given prognostic factor in the total sample (k = 105).

eTable 7. Frequency of not reported data on each prognostic risk factor in four therapy submodalities

	Therapy submodality				
Prognostic risk factor	Total<br>No. (%)	Ind FTF<br>No. (%)	Group FTF<br>No. (%)	Guided<br>IBT<br>No. (%)	Self-guided<br>IBT<br>No. (%)
Symptom severity	35 (33)	3 (13.6)	15 (57.7)	6 (25.0)	9 (36.0)
Diagnosis	9 (9)	1 (4.5)	1 (3.8)	2 (8.3)	3 (12.0)
Chronic or recurrent depression	59 (56)	9 (40.9)	13 (50.0)	12 (50.0)	19 (76.0)
Comorbid mental disorders	65 (62)	5 (22.7)	16 (61.5)	19 (79.2)	21 (84.0)
Comorbid personality disorders	92 (87)	14 (63.6)	21 (80.8)	24 (100)	25 (100)
Comorbid substance use disorder	51 (48)	6 (27.3)	9 (34.6)	15 (62.5)	18 (72.0)
No college	27 (26)	5 (22.7)	6 (23.1)	3 (12.5)	12 (48.0)
Low income	90 (86)	17 (77.3)	24 (92.3)	18 (75.0)	24 (96.0)
No occupation	65 (62)	15 (68.2)	17 (65.4)	11 (45.8)	16 (64.0)
Ethnic minority	76 (72)	11 (50.0)	22 (84.6)	21 (87.5)	15 (60.0)
Single/no partner	61 (58)	16 (72.7)	16 (61.5)	10 (41.7)	15 (60.0)
Age > 65 years	36 (34)	4 (18.2)	6 (23.1)	12 (50.0)	11 (44.0)

Abbreviations: Ind FTF, individual face-to-face therapy (k = 22); Group FTF, group face-to-face therapy (k = 26); Guided IBT, guided internet-based therapy (k = 24); Self-guided IBT, self-guided internet-based therapy (k = 25).

eTable 8. Extract from the codebook with definitions of prognostic risk factors[a]

Prognostic risk factors	Definition	References
Symptom severity	Screening instruments not mentioned hereafter can be used if cut-offs for moderate and mild depression are availabe<br>1 = All patients at least moderate depression: PHQ-9 ≥ 11, BDI-I ≥ 15, BDI-II ≥ 20, CES-D ≥ 26, Ham-D ≥ 13<br>0.5 = All patients at least mild depression: PHQ-9 ≥ 5, BDI-I ≥ 9, BDI-II ≥ 10, CES-D ≥ 14, Ham-D ≥ 9<br>0 = Cut-off for study entry was less than mild depression<br>99 = Insufficient information	Beutler LE, Castonguay LG, Follette WC. Therapeutic factors in dysphoric disorders. *J Clin Psychol.* 2006;62(6):639–647. doi:10.1002/jclp.20260.<br><br>Bower P, Kontopantelis E, Sutton A, et al. Influence of initial severity of depression on effectiveness of low intensity interventions: meta-analysis of individual patient data. *BMJ.* 2013;346:f540. doi:10.1136/bmj.f540.<br><br>Buckman JEJ, Saunders R, Cohen ZD, et al. The contribution of depressive 'disorder characteristics' to determinations of prognosis for adults with depression: an individual patient data meta-analysis. *Psychol Med.* 2021;51(7):1068–1081. doi:10.1017/S0033291721001367.<br><br>Garfield SL, Bergin AE. Bergin and Garfield's handbook of psychotherapy and behavior change. 50th ed. Hoboken, NJ: John Wiley & Sons; 2021.
Clinical diagnosis of depression	1 = Included patients have been diagnosed with a depressive disorder based on a clinical interview. Clinical diagnoses and thresholds on depression scales are not accepted as diagnosis for this item<br>0.5 = Diagnoses from clinical routine<br>0 = Patients have not been diagnosed with a depressive disorder based on a clinical interview<br>99 = Insufficient information	
Chronicity of depression	1 = At least 50% of the included patients have a history of depression or are diagnosed with a chronic persistent depressive disorder<br>0.5 = At least 33% but less than 50% of included patients have a history of depression or are diagnosed with a chronic persistent depressive disorder<br>0 = Less than 33% of the included patients have a history of depression or are diagnosed with a chronic persistent depressive disorder	Buckman JEJ, Saunders R, Cohen ZD, et al. The contribution of depressive 'disorder characteristics' to determinations of prognosis for adults with depression: an individual patient data meta-analysis. *Psychol Med.* 2021;51(7):1068–1081. doi:10.1017/S0033291721001367.

© 2023 Merzhvynska M et al. *JAMA Psychiatry.*

	99 = Insufficient information	
Comorbid mental disorders	Relevant comorbid mental disorders include classes of mental disorders except organic mental disorders, personality disorders, and substance abuse disorders<br>1 = At least 50% of included patients had a comorbid mental disorder<br>0.5 = At least 33% but less than 50% of included patients had a comorbid mental disorder<br>0 = Less than 33% of included patients had a comorbid mental disorder<br>99 = Insufficient information	Buckman JEJ, Saunders R, Cohen ZD, et al. The contribution of depressive 'disorder characteristics' to determinations of prognosis for adults with depression: an individual patient data meta-analysis. *Psychol Med.* 2021;51(7):1068–1081. doi:10.1017/S0033291721001367.<br><br>van Beljouw IM, Verhaak PF, Cuijpers P, van Marwijk HW, Penninx BW. The course of untreated anxiety and depression, and determinants of poor one-year outcome: a one-year cohort study. *BMC Psychiatry.* 2010;10:86. doi:10.1186/1471-244X-10-86.
Comorbid personality disorder	Relevant comorbid personality disorders included all personality disorders except antisocial personality disorder<br>1 = Patients with comorbid personality disorders were allowed to enter the trial and some included patients had a comorbid personality disorder<br>0.5 = Some but not all personality disorders are excluded and some included patients had a comorbid personality disorder<br>0 = Patients with comorbid Axis II disorders were not allowed to enter the trial or none of the included patients had a comorbid personality disorder<br>99 = Insufficient information to make the determination	Beutler LE, Castonguay LG, Follette WC. Therapeutic factors in dysphoric disorders. *J Clin Psychol.* 2006;62(6):639–647. doi:10.1002/jclp.20260.<br><br>Reich J. The effect of Axis II disorders on the outcome of treatment of anxiety and unipolar depressive disorders: a review. *J Pers Disord.* 2003;17(5):387–405. doi:10.1521/pedi.17.5.387.22972.
Comorbid substance use disorders	1 = Patients with comorbid substance use disorder were allowed to enter the trial and some of included patients had a comorbid substance use disorder<br>0 = Patients with comorbid substance use disorder were not allowed to enter the trial or none of the included patients had a substance use disorder<br>99 = Insufficient information	Hasin DS, Tsai WY, Endicott J, Mueller TI, Coryell W, Keller M. Five-year course of major depression: effects of comorbid alcoholism. *J Affect Disord.* 1996;41(1):63–70. doi:10.1016/0165-0327(96)00068-7.<br><br>Agosti V, Levin FR. The effects of alcohol and drug dependence on the course of depression. *Am J Addict.* 2006;15(1):71–75. doi:10.1080/10550490500419102.

© 2023 Merzhvynska M et al. *JAMA Psychiatry.*

No partner	1 = At least 50% of included patients have no partner<br>0.5 = At least 33 but less than 50% of included patients have no partner<br>0 = Less than 33% of included patients have no partner<br>99 = Insufficient information	Buckman JEJ, Saunders R, Stott J, et al. Role of age, gender and marital status in prognosis for adults with depression: An individual patient data meta-analysis. *Epidemiol Psychiatr Sci.* 2021;30:e42. doi:10.1017/S2045796021000342.
Lower education	Lower education was defined as not having some college education<br>1 = At least 50% of included patients have no college education<br>0.5 = At least 33% but less than 50% of included patients have no college education<br>0 = Less than 33% of included patients have no college education<br>99 = Insufficient information	Eaton WW, Shao H, Nestadt G, Lee HB, Bienvenu OJ, Zandi P. Population-based study of first onset and chronicity in major depressive disorder. *Arch Gen Psychiatry.* 2008;65(5):513–520. doi:10.1001/archpsyc.65.5.513.
Lower income	1 = At least 20% of included patients have lower income<br>0 = Less than 20% of included patients have lower income<br>99 = Insufficient information	Falconnier L. Socioeconomic status in the treatment of depression. *Am J Orthopsychiatry.* 2009;79(2):148–158. doi:10.1037/a0015469.
No occupation	1 = At least 20% of included patients are unemployed or disabled<br>0.5 = At least 12.5% but less than 20% of included patients are unemployed or disabled<br>0 = Less than 12.5% of included patients are unemployed or disabled<br>99 = Insufficient information	Buckman JEJ, Saunders R, Stott J, et al. Socioeconomic Indicators of Treatment Prognosis for Adults With Depression: A Systematic Review and Individual Patient Data Meta-analysis. *JAMA Psychiatry.* 2022;79(5):406–416. doi:10.1001/jamapsychiatry.2022.0100.<br><br>Delgadillo J, Moreea O, Lutz W. Different people respond differently to therapy: A demonstration using patient profiling and risk stratification. *Behav Res Ther.* 2016;79:15–22. doi:10.1016/j.brat.2016.02.003.
Ethnic minority	1 = At least 20% of included patients belong to an ethnic minority group<br>0.5 = At least 12.5 but less than 20% of included patients belong to an ethnic minority group<br>0 = Less than 12.5% of included patients belong to an ethnic minority group<br>99 = Insuficient information	Beutler LE, Castonguay LG, Follette WC. Therapeutic factors in dysphoric disorders. *J Clin Psychol.* 2006;62(6):639–647. doi:10.1002/jclp.20260.

© 2023 Merzhvynska M et al. *JAMA Psychiatry.*

Older age	1 = Some of the included patients are older than 65<br>0 = Patients older than 65 are not included in the trial<br>99 = Insufficient information	Fournier JC, DeRubeis RJ, Shelton RC, Hollon SD, Amsterdam JD, Gallop R. Prediction of response to medication and cognitive therapy in the treatment of moderate to severe depression. *J Consult Clin Psychol.* 2009;77(4):775–787. doi:10.1037/a0015401.<br><br>Thase ME, Greenhouse JB, Frank E, et al. Treatment of major depression with psychotherapy or psychotherapy-pharmacotherapy combinations. *Arch Gen Psychiatry.* 1997;54(11):1009–1015. doi:10.1001/archpsyc.1997.01830230043006.

[a] The codebook with a description of all variables can be found at OSF https://osf.io/wt8fb

© 2023 Merzhvynska M et al. *JAMA Psychiatry.*

eReferences 1. Trials included in the systematic review contrasting face-to-face and internet-based therapy for depression

References marked with ‡ denote four reports of trials with outlying effect sizes (Hedges' *g* > 2.0).

1. Addington EL, Cheung EO, Bassett SM, et al. The MARIGOLD study: Feasibility and enhancement of an online intervention to improve emotion regulation in people with elevated depressive symptoms. *J Affect Disord.* 2019;257:352–364. doi:10.1016/j.jad.2019.07.049.

2. Andersson G, Bergström J, Holländare F, Carlbring P, Kaldo V, Ekselius L. Internet-based self-help for depression: randomised controlled trial. *Br J Psychiatry.* 2005;187:456–461. doi:10.1192/bjp.187.5.456.

3. Anuwatgasem C, Awirutworakul T, Vallibhakara SA-O, et al. The Effects of Mindfulness and Self-Compassion-Based Group Therapy for Major Depressive Disorder: A Randomized Controlled Trial. *JOURNAL OF THE MEDICAL ASSOCIATION OF THAILAND.* 2020;103(9):856–863.

4. Arroll B, Frischtak H, Roskvist R, et al. FACT effectiveness in primary care; a single visit RCT for depressive symptoms. *Int J Psychiatry Med.* 2022;57(2):91–102. doi:10.1177/00912174211010536.

5. Barnhofer T, Crane C, Hargus E, Amarasinghe M, Winder R, Williams JMG. Mindfulness-based cognitive therapy as a treatment for chronic depression: A preliminary study. *Behav Res Ther.* 2009;47(5):366–373. doi:10.1016/j.brat.2009.01.019.

6. Beevers CG, Pearson R, Hoffman JS, Foulser AA, Shumake J, Meyer B. Effectiveness of an internet intervention (Deprexis) for depression in a united states adult sample: A parallel-group pragmatic randomized controlled trial. *J Consult Clin Psychol.* 2017;85(4):367–380. doi:10.1037/ccp0000171.

7. Berger T, Hämmerli K, Gubser N, Andersson G, Caspar F. Internet-based treatment of depression: a randomized controlled trial comparing guided with unguided self-help. *Cogn Behav Ther.* 2011;40(4):251–266. doi:10.1080/16506073.2011.616531.

8. Berking M, Eichler E, Luhmann M, Diedrich A, Hiller W, Rief W. Affect regulation training reduces symptom severity in depression - A randomized controlled trial. *PLoS One.* 2019;14(8):e0220436. doi:10.1371/journal.pone.0220436.

9. Bohlmeijer ET, Fledderus M, Rokx TAJJ, Pieterse ME. Efficacy of an early intervention based on acceptance and commitment therapy for adults with depressive symptomatology: Evaluation in a randomized controlled trial. *Behav Res Ther.* 2011;49(1):62–67. doi:10.1016/j.brat.2010.10.003.

10. Bolier L, Haverman M, Kramer J, et al. An Internet-based intervention to promote mental fitness for mildly depressed adults: randomized controlled trial. *J Med Internet Res.* 2013;15(9):e200. doi:10.2196/jmir.2603.

11. Bücker L, Schnakenberg P, Karyotaki E, Moritz S, Westermann S. Diminishing Effects After Recurrent Use of Self-Guided Internet-Based Interventions in Depression: Randomized Controlled Trial. *J Med Internet Res.* 2019;21(10):e14240. doi:10.2196/14240.

12. Buntrock C, Ebert D, Lehr D, et al. Effectiveness of a web-based cognitive behavioural intervention for subthreshold depression: pragmatic randomised controlled trial. *Psychother Psychosom.* 2015;84(6):348–358. doi:10.1159/000438673.

13. Carlbring P, Hägglund M, Luthström A, et al. Internet-based behavioral activation and acceptance-based treatment for depression: a randomized controlled trial. *J Affect Disord.* 2013;148(2-3):331–337. doi:10.1016/j.jad.2012.12.020.

© 2023 Merzhvynska M et al. *JAMA Psychiatry.*

14. Carr A, Finnegan L, Griffin E, Cotter P, Hyland A. A Randomized Controlled Trial of the Say Yes to Life (SYTL) Positive Psychology Group Psychotherapy Program for Depression: An Interim Report. *J Contemp Psychother.* 2017;47(3):153–161. doi:10.1007/s10879-016-9343-6.

15. Carta M, Petretto D, Adamo S, et al. Counseling in primary care improves depression and quality of life. *Clin Pract Epidemiol Ment Health.* 2012;8:152–157. doi:10.2174/1745017901208010152.

16. Castonguay LG, Schut AJ, Aikens DE, et al. Integrative Cognitive Therapy for Depression: A Preliminary Investigation. *Journal of Psychotherapy Integration.* 2004;14(1):4–20. doi:10.1037/1053-0479.14.1.4.

17. Chan AS, Wong QY, Sze SL, Kwong PPK, Han YMY, Cheung M-C. A Chinese Chan-based mind-body intervention for patients with depression. *J Affect Disord.* 2012;142(1-3):283–289. doi:10.1016/j.jad.2012.05.018.

18. ‡ Chiang K-J, Chen T-H, Hsieh H-T, Tsai J-C, Ou K-L, Chou K-R. One-Year Follow-Up of the Effectiveness of Cognitive Behavioral Group Therapy for Patients' Depression: A Randomized, Single-Blinded, Controlled Study. *ScientificWorldJournal.* 2015;2015:373149. doi:10.1155/2015/373149.

19. Cladder-Micus MB, Speckens AEM, Vrijsen JN, T Donders AR, Becker ES, Spijker J. Mindfulness-based cognitive therapy for patients with chronic, treatment-resistant depression: A pragmatic randomized controlled trial. *Depress Anxiety.* 2018;35(10):914–924. doi:10.1002/da.22788.

20. Clarke G, Eubanks D, Reid E, et al. Overcoming Depression on the Internet (ODIN) (2): a randomized trial of a self-help depression skills program with reminders. *J Med Internet Res.* 2005;7(2):e16. doi:10.2196/jmir.7.2.e16.

21. Clarke G, Reid E, Eubanks D, et al. Overcoming depression on the Internet (ODIN): a randomized controlled trial of an Internet depression skills intervention program. *J Med Internet Res.* 2002;4(3):E14. doi:10.2196/jmir.4.3.e14.

22. Dahne J, Lejuez CW, Diaz VA, et al. Pilot Randomized Trial of a Self-Help Behavioral Activation Mobile App for Utilization in Primary Care. *Behav Ther.* 2019;50(4):817–827. doi:10.1016/j.beth.2018.12.003.

23. Ebert DD, Buntrock C, Lehr D, et al. Effectiveness of Web- and Mobile-Based Treatment of Subthreshold Depression With Adherence-Focused Guidance: A Single-Blind Randomized Controlled Trial. *Behav Ther.* 2018;49(1):71–83. doi:10.1016/j.beth.2017.05.004.

24. ‡ Embling S. The effectiveness of cognitive behavioural therapy in depression. *Nurs Stand.* 2002;17(14-15):33–41. doi:10.7748/ns2002.12.17.14.33.c3318.

25. Everitt N, Broadbent J, Richardson B, et al. Exploring the features of an app-based just-in-time intervention for depression. *J Affect Disord.* 2021;291:279–287. doi:10.1016/j.jad.2021.05.021.

26. Farrer L, Christensen H, Griffiths KM, Mackinnon A. Internet-based CBT for depression with and without telephone tracking in a national helpline: randomised controlled trial. *PLoS One.* 2011;6(11):e28099. doi:10.1371/journal.pone.0028099.

27. Fonagy P, Rost F, Carlyle J-A, et al. Pragmatic randomized controlled trial of long-term psychoanalytic psychotherapy for treatment-resistant depression: the Tavistock Adult Depression Study (TADS). *World Psychiatry.* 2015;14(3):312–321. doi:10.1002/wps.20267.

28. Forand NR, Barnett JG, Strunk DR, Hindiyeh MU, Feinberg JE, Keefe JR. Efficacy of Guided iCBT for Depression and Mediation of Change by Cognitive Skill Acquisition. *Behav Ther.* 2018;49(2):295–307. doi:10.1016/j.beth.2017.04.004.

29. Fuhr K, Fahse B, Hautzinger M, Gulewitsch MD. Erste Erfahrungen zur Implementierbarkeit einer internet-basierten Selbsthilfe zur Überbrückung der Wartezeit auf eine ambulante Psychotherapie. *Psychother Psychosom Med Psychol.* 2018;68(6):234–241. doi:10.1055/s-0043-122241.

© 2023 Merzhvynska M et al. *JAMA Psychiatry.*

30. Geraedts AS, Kleiboer AM, Wiezer NM, van Mechelen W, Cuijpers P. Short-term effects of a web-based guided self-help intervention for employees with depressive symptoms: randomized controlled trial. *J Med Internet Res.* 2014;16(5):e121. doi:10.2196/jmir.3185.

31. Gibbons MBC, Thompson SM, Scott K, et al. Supportive-expressive dynamic psychotherapy in the community mental health system: a pilot effectiveness trial for the treatment of depression. *Psychotherapy (Chic).* 2012;49(3):303–316. doi:10.1037/a0027694.

32. Gilbody S, Littlewood E, Hewitt C, et al. Computerised cognitive behaviour therapy (cCBT) as treatment for depression in primary care (REEACT trial): large scale pragmatic randomised controlled trial. *BMJ.* 2015;351:h5627. doi:10.1136/bmj.h5627.

33. Graaf LE de, Gerhards SAH, Arntz A, et al. Clinical effectiveness of online computerised cognitive-behavioural therapy without support for depression in primary care: randomised trial. *Br J Psychiatry.* 2009;195(1):73–80. doi:10.1192/bjp.bp.108.054429.

34. Gräfe V, Moritz S, Greiner W. Health economic evaluation of an internet intervention for depression (deprexis), a randomized controlled trial. *Health Econ Rev.* 2020;10(1):19. doi:10.1186/s13561-020-00273-0.

35. ‡ Hagen R, Hjemdal O, Solem S, et al. Metacognitive Therapy for Depression in Adults: A Waiting List Randomized Controlled Trial with Six Months Follow-Up. *Front. Psychol.* 2017;8. doi:10.3389/fpsyg.2017.00031.

36. Hallgren M, Kraepelien M, Öjehagen A, et al. Physical exercise and internet-based cognitive-behavioural therapy in the treatment of depression: randomised controlled trial. *Br J Psychiatry.* 2015;207(3):227–234. doi:10.1192/bjp.bp.114.160101.

37. Harley R, Sprich S, Safren S, Jacobo M, Fava M. Adaptation of dialectical behavior therapy skills training group for treatment-resistant depression. *J Nerv Ment Dis.* 2008;196(2):136–143. doi:10.1097/NMD.0b013e318162aa3f.

38. Hemanny C, Carvalho C, Maia N, et al. Efficacy of trial-based cognitive therapy, behavioral activation and treatment as usual in the treatment of major depressive disorder: preliminary findings from a randomized clinical trial. *CNS Spectr.* 2020;25(4):535–544. doi:10.1017/S1092852919001457.

39. Holas P, Krejtz I, Wisiecka K, Rusanowska M, Nezlek JB. Modification of Attentional Bias to Emotional Faces Following Mindfulness-Based Cognitive Therapy in People with a Current Depression. *Mindfulness.* 2020;11(6):1413–1423. doi:10.1007/s12671-020-01353-2.

40. Jelinek L, Arlt S, Moritz S, Schröder J, Westermann S, Cludius B. Brief Web-Based Intervention for Depression: Randomized Controlled Trial on Behavioral Activation. *J Med Internet Res.* 2020;22(3):e15312. doi:10.2196/15312.

41. Johansson O, Bjärehed J, Andersson G, Carlbring P, Lundh L-G. Effectiveness of guided internet-delivered cognitive behavior therapy for depression in routine psychiatry: A randomized controlled trial. *Internet Interv.* 2019;17:100247. doi:10.1016/j.invent.2019.100247.

42. Jurinec N, Schienle A. Utilizing placebos to leverage effects of cognitive-behavioral therapy in patients with depression. *J Affect Disord.* 2020;277:779–784. doi:10.1016/j.jad.2020.08.087.

43. Kenter RMF, Cuijpers P, Beekman A, van Straten A. Effectiveness of a Web-Based Guided Self-help Intervention for Outpatients With a Depressive Disorder: Short-term Results From a Randomized Controlled Trial. *J Med Internet Res.* 2016;18(3):e80. doi:10.2196/jmir.4861.

44. King M, Sibbald B, Ward E, et al. Randomised controlled trial of non-directive counselling, cognitive-behaviour therapy and usual general practitioner care in the management of depression as well as mixed anxiety and depression in primary care. *Health Technol Assess.* 2000;4(19):1–83.

© 2023 Merzhvynska M et al. *JAMA Psychiatry.*

45. Kivi M, Eriksson MCM, Hange D, et al. Internet-based therapy for mild to moderate depression in Swedish primary care: short term results from the PRIM-NET randomized controlled trial. *Cogn Behav Ther.* 2014;43(4):289–298. doi:10.1080/16506073.2014.921834.

46. Kleiboer A, Donker T, Seekles W, van Straten A, Riper H, Cuijpers P. A randomized controlled trial on the role of support in Internet-based problem solving therapy for depression and anxiety. *Behav Res Ther.* 2015;72:63–71. doi:10.1016/j.brat.2015.06.013.

47. Klein JP, Berger T, Schröder J, et al. Effects of a Psychological Internet Intervention in the Treatment of Mild to Moderate Depressive Symptoms: Results of the EVIDENT Study, a Randomized Controlled Trial. *Psychother Psychosom.* 2016;85(4):218–228. doi:10.1159/000445355.

48. Krämer LV, Grünzig S-D, Baumeister H, Ebert DD, Bengel J. Effectiveness of a Guided Web-Based Intervention to Reduce Depressive Symptoms before Outpatient Psychotherapy: A Pragmatic Randomized Controlled Trial. *Psychother Psychosom.* 2021;90(4):233–242. doi:10.1159/000515625.

49. Lambert JD, Greaves CJ, Farrand P, Price L, Haase AM, Taylor AH. Web-Based Intervention Using Behavioral Activation and Physical Activity for Adults With Depression (The eMotion Study): Pilot Randomized Controlled Trial. *J Med Internet Res.* 2018;20(7):e10112. doi:10.2196/10112.

50. Lappalainen P, Langrial S, Oinas-Kukkonen H, Tolvanen A, Lappalainen R. Web-based acceptance and commitment therapy for depressive symptoms with minimal support: a randomized controlled trial. *Behav Modif.* 2015;39(6):805–834. doi:10.1177/0145445515598142.

51. Lee E, Han Y, Cha YJ, et al. Community-Based Multi-Site Randomized Controlled Trial of Behavioral Activation for Patients with Depressive Disorders. *Community Ment Health J.* 2021;58(2):343–355. doi:10.1007/s10597-021-00828-3.

52. Lemma A, Fonagy P. Feasibility study of a psychodynamic online group intervention for depression. *Psychoanalytic Psychology.* 2013;30(3):367–380. doi:10.1037/a0033239.

53. Löbner M, Pabst A, Stein J, et al. Computerized cognitive behavior therapy for patients with mild to moderately severe depression in primary care: A pragmatic cluster randomized controlled trial (@ktiv). *J Affect Disord.* 2018;238:317–326. doi:10.1016/j.jad.2018.06.008.

54. Lüdtke T, Pult LK, Schröder J, Moritz S, Bücker L. A randomized controlled trial on a smartphone self-help application (Be Good to Yourself) to reduce depressive symptoms. *Psychiatry Res.* 2018;269:753–762. doi:10.1016/j.psychres.2018.08.113.

55. Lynch TR, Hempel RJ, Whalley B, et al. Refractory depression - mechanisms and efficacy of radically open dialectical behaviour therapy (RefraMED): findings of a randomised trial on benefits and harms. *Br J Psychiatry.* 2020;216(4):204–212. doi:10.1192/bjp.2019.53.

56. MacLean S, Corsi DJ, Litchfield S, et al. Coach-Facilitated Web-Based Therapy Compared With Information About Web-Based Resources in Patients Referred to Secondary Mental Health Care for Depression: Randomized Controlled Trial. *J Med Internet Res.* 2020;22(6):e15001. doi:10.2196/15001.

57. MacPherson H, Richmond S, Bland M, et al. Acupuncture and counselling for depression in primary care: a randomised controlled trial. *PLoS Med.* 2013;10(9):e1001518. doi:10.1371/journal.pmed.1001518.

58. Mahmoodi M, Bakhtiyari M, Masjedi Arani A, Mohammadi A, Saberi Isfeedvajani M. The comparison between CBT focused on perfectionism and CBT focused on emotion regulation for individuals with depression and anxiety disorders and dysfunctional perfectionism: a randomized controlled trial. *Behav Cogn Psychother.* 2020:1–18. doi:10.1017/S1352465820000909.

© 2023 Merzhvynska M et al. *JAMA Psychiatry.*

59. Maina G, Forner F, Bogetto F. Randomized controlled trial comparing brief dynamic and supportive therapy with waiting list condition in minor depressive disorders. *Psychother Psychosom.* 2005;74(1):43–50. doi:10.1159/000082026.

60. Meyer B, Berger T, Caspar F, Beevers CG, Andersson G, Weiss M. Effectiveness of a novel integrative online treatment for depression (Deprexis): randomized controlled trial. *J Med Internet Res.* 2009;11(2):e15. doi:10.2196/jmir.1151.

61. Meyer B, Bierbrodt J, Schröder J, et al. Effects of an Internet intervention (Deprexis) on severe depression symptoms: Randomized controlled trial. *Internet Interv.* 2015;2(1):48–59. doi:10.1016/j.invent.2014.12.003.

62. Michalak J, Schultze M, Heidenreich T, Schramm E. A randomized controlled trial on the efficacy of mindfulness-based cognitive therapy and a group version of cognitive behavioral analysis system of psychotherapy for chronically depressed patients. *J Consult Clin Psychol.* 2015;83(5):951–963. doi:10.1037/ccp0000042.

63. Mira A, Bretón-López J, García-Palacios A, Quero S, Baños RM, Botella C. An Internet-based program for depressive symptoms using human and automated support: a randomized controlled trial. *Neuropsychiatr Dis Treat.* 2017;13:987–1006. doi:10.2147/NDT.S130994.

64. Mohr DC, Duffecy J, Ho J, et al. A randomized controlled trial evaluating a manualized TeleCoaching protocol for improving adherence to a web-based intervention for the treatment of depression. *PLoS One.* 2013;8(8):e70086. doi:10.1371/journal.pone.0070086.

65. Montero-Marín J, Araya R, Pérez-Yus MC, et al. An Internet-Based Intervention for Depression in Primary Care in Spain: A Randomized Controlled Trial. *J Med Internet Res.* 2016;18(8):e231. doi:10.2196/jmir.5695.

66. Moritz S, Schilling L, Hauschildt M, Schröder J, Treszl A. A randomized controlled trial of internet-based therapy in depression. *Behav Res Ther.* 2012;50(7-8):513–521. doi:10.1016/j.brat.2012.04.006.

67. ‡ Mukhtar F, Oei TPS. Predictors of Group Cognitive Behaviour Therapy outcomes for the treatment of depression in Malaysia. *Asian J Psychiatr.* 2011;4(2):125–128. doi:10.1016/j.ajp.2011.04.002.

68. Naeem F, Gul M, Irfan M, et al. Brief culturally adapted CBT (CaCBT) for depression: a randomized controlled trial from Pakistan. *J Affect Disord.* 2015;177:101–107. doi:10.1016/j.jad.2015.02.012.

69. Niedermoser DW, Kalak N, Kiyhankhadiv A, et al. Workplace-Related Interpersonal Group Psychotherapy to Improve Life at Work in Individuals With Major Depressive Disorders: A Randomized Interventional Pilot Study. *Front Psychiatry.* 2020;11:168. doi:10.3389/fpsyt.2020.00168.

70. Nyström MBT, Stenling A, Sjöström E, et al. Behavioral activation versus physical activity via the internet: A randomized controlled trial. *J Affect Disord.* 2017;215:85–93. doi:10.1016/j.jad.2017.03.018.

71. Omidi A, Mohammadkhani P, Mohammadi A, Zargar F. Comparing mindfulness based cognitive therapy and traditional cognitive behavior therapy with treatments as usual on reduction of major depressive disorder symptoms. *Iran Red Crescent Med J.* 2013;15(2):142–146. doi:10.5812/ircmj.8018.

72. Perini S, Titov N, Andrews G. Clinician-assisted Internet-based treatment is effective for depression: randomized controlled trial. *Aust N Z J Psychiatry.* 2009;43(6):571–578. doi:10.1080/00048670902873722.

73. Pots WTM, Fledderus M, Meulenbeek PAM, Klooster PM ten, Schreurs KMG, Bohlmeijer ET. Acceptance and commitment therapy as a web-based intervention for depressive symptoms: randomised controlled trial. *Br J Psychiatry.* 2016;208(1):69–77. doi:10.1192/bjp.bp.114.146068.

74. Pots WTM, Meulenbeek PAM, Veehof MM, Klungers J, Bohlmeijer ET. The efficacy of mindfulness-based cognitive therapy as a public mental health intervention for adults with mild to moderate depressive symptomatology: a randomized controlled trial. *PLoS One.* 2014;9(10):e109789. doi:10.1371/journal.pone.0109789.

75. Richards D, Timulak L, O'Brien E, et al. A randomized controlled trial of an internet-delivered treatment: Its potential as a low-intensity community intervention for adults with symptoms of depression. *Behav Res Ther.* 2015;75:20–31. doi:10.1016/j.brat.2015.10.005.

76. Rief W, Bleichhardt G, Dannehl K, Euteneuer F, Wambach K. Comparing the Efficacy of CBASP with Two Versions of CBT for Depression in a Routine Care Center: A Randomized Clinical Trial. *Psychother Psychosom.* 2018;87(3):164–178. doi:10.1159/000487893.

77. Roepke AM, Jaffee SR, Riffle OM, McGonigal J, Broome R, Maxwell B. Randomized Controlled Trial of SuperBetter, a Smartphone-Based/Internet-Based Self-Help Tool to Reduce Depressive Symptoms. *Games Health J.* 2015;4(3):235–246. doi:10.1089/g4h.2014.0046.

78. Röhricht F, Papadopoulos N, Priebe S. An exploratory randomized controlled trial of body psychotherapy for patients with chronic depression. *J Affect Disord.* 2013;151(1):85–91. doi:10.1016/j.jad.2013.05.056.

79. Rosso IM, Killgore WDS, Olson EA, et al. Internet-based cognitive behavior therapy for major depressive disorder: A randomized controlled trial. *Depress Anxiety.* 2017;34(3):236–245. doi:10.1002/da.22590.

80. Ruwaard J, Schrieken B, Schrijver M, et al. Standardized web-based cognitive behavioural therapy of mild to moderate depression: a randomized controlled trial with a long-term follow-up. *Cogn Behav Ther.* 2009;38(4):206–221. doi:10.1080/16506070802408086.

81. Schramm E, Mack S, Thiel N, Jenkner C, Elsaesser M, Fangmeier T. Interpersonal Psychotherapy vs. Treatment as Usual for Major Depression Related to Work Stress: A Pilot Randomized Controlled Study. *Front Psychiatry.* 2020;11:193. doi:10.3389/fpsyt.2020.00193.

82. Schuling R, Huijbers MJ, van Ravesteijn H, et al. Recovery from recurrent depression: Randomized controlled trial of the efficacy of mindfulness-based compassionate living compared with treatment-as-usual on depressive symptoms and its consolidation at longer term follow-up. *J Affect Disord.* 2020;273:265–273. doi:10.1016/j.jad.2020.03.182.

83. Schure MB, Lindow JC, Greist JH, et al. Use of a Fully Automated Internet-Based Cognitive Behavior Therapy Intervention in a Community Population of Adults With Depression Symptoms: Randomized Controlled Trial. *J Med Internet Res.* 2019;21(11):e14754. doi:10.2196/14754.

84. Schuster R, Leitner I, Carlbring P, Laireiter A-R. Exploring blended group interventions for depression: Randomised controlled feasibility study of a blended computer- and multimedia-supported psychoeducational group intervention for adults with depressive symptoms. *Internet Interv.* 2017;8:63–71. doi:10.1016/j.invent.2017.04.001.

85. Segal ZV, Dimidjian S, Beck A, et al. Outcomes of Online Mindfulness-Based Cognitive Therapy for Patients With Residual Depressive Symptoms: A Randomized Clinical Trial. *JAMA Psychiatry.* 2020;77(6):563–573. doi:10.1001/jamapsychiatry.2019.4693.

86. Seligman MEP, Rashid T, Parks AC. Positive psychotherapy. *Am Psychol.* 2006;61(8):774–788. doi:10.1037/0003-066X.61.8.774.

87. Sinniah A, Oei TPS, Maniam T, Subramaniam P. Positive effects of Individual Cognitive Behavior Therapy for patients with unipolar mood disorders with suicidal ideation in Malaysia: A randomised controlled trial. *Psychiatry Res.* 2017;254:179–189. doi:10.1016/j.psychres.2017.04.026.

88. Smith J, Newby JM, Burston N, et al. Help from home for depression: A randomised controlled trial comparing internet-delivered cognitive behaviour therapy with bibliotherapy for depression. *Internet Interv.* 2017;9:25–37. doi:10.1016/j.invent.2017.05.001.

89. Stiles-Shields C, Montague E, Kwasny MJ, Mohr DC. Behavioral and cognitive intervention strategies delivered via coached apps for depression: Pilot trial. *Psychol Serv.* 2019;16(2):233–238. doi:10.1037/ser0000261.

90. Strauss C, Hayward M, Chadwick P. Group person-based cognitive therapy for chronic depression: a pilot randomized controlled trial. *Br J Clin Psychol.* 2012;51(3):345–350. doi:10.1111/j.2044-8260.2012.02036.x.

91. Sugg HVR, Frost J, Richards DA. Morita Therapy for depression (Morita Trial): an embedded qualitative study of acceptability. *BMJ Open.* 2018;9(5):e023873. doi:10.1136/bmjopen-2018-023873.

92. Szumska I, Gola M, Rusanowska M, et al. Mindfulness-based cognitive therapy reduces clinical symptoms, but do not change frontal alpha asymmetry in people with major depression disorder. *Int J Neurosci.* 2021;131(5):453–461. doi:10.1080/00207454.2020.1748621.

93. Titov N, Andrews G, Davies M, McIntyre K, Robinson E, Solley K. Internet treatment for depression: a randomized controlled trial comparing clinician vs. technician assistance. *PLoS One.* 2010;5(6):e10939. doi:10.1371/journal.pone.0010939.

94. Tong P, Bu P, Yang Y, Dong L, Sun T, Shi Y. Group cognitive behavioural therapy can reduce stigma and improve treatment compliance in major depressive disorder patients. *Early Interv Psychiatry.* 2020;14(2):172–178. doi:10.1111/eip.12841.

95. Town JM, Abbass A, Stride C, Bernier D. A randomised controlled trial of Intensive Short-Term Dynamic Psychotherapy for treatment resistant depression: the Halifax Depression Study. *J Affect Disord.* 2017;214:15–25. doi:10.1016/j.jad.2017.02.035.

96. Tulbure BT, Andersson G, Sălăgean N, Pearce M, Koenig HG. Religious versus Conventional Internet-based Cognitive Behavioral Therapy for Depression. *J Relig Health.* 2018;57(5):1634–1648. doi:10.1007/s10943-017-0503-0.

97. Vernmark K, Lenndin J, Bjärehed J, et al. Internet administered guided self-help versus individualized e-mail therapy: A randomized trial of two versions of CBT for major depression. *Behav Res Ther.* 2010;48(5):368–376. doi:10.1016/j.brat.2010.01.005.

98. Warmerdam L, van Straten A, Twisk J, Riper H, Cuijpers P. Internet-based treatment for adults with depressive symptoms: randomized controlled trial. *J Med Internet Res.* 2008;10(4):e44. doi:10.2196/jmir.1094.

99. Wiersma JE, van Schaik DJF, Hoogendorn AW, et al. The effectiveness of the cognitive behavioral analysis system of psychotherapy for chronic depression: a randomized controlled trial. *Psychother Psychosom.* 2014;83(5):263–269. doi:10.1159/000360795.

100. Wiles N, Thomas L, Abel A, et al. Cognitive behavioural therapy as an adjunct to pharmacotherapy for primary care based patients with treatment resistant depression: results of the CoBalT randomised controlled trial. *Lancet.* 2013;381(9864):375–384. doi:10.1016/S0140-6736(12)61552-9.

101. Williams AD, Blackwell SE, Mackenzie A, Holmes EA, Andrews G. Combining imagination and reason in the treatment of depression: a randomized controlled trial of internet-based cognitive-bias modification and internet-CBT for depression. *J Consult Clin Psychol.* 2013;81(5):793–799. doi:10.1037/a0033247.

102. Wong DFK. Cognitive behavioral treatment groups for people with chronic depression in Hong Kong: a randomized wait-list control design. *Depress Anxiety.* 2008;25(2):142–148. doi:10.1002/da.20286.

© 2023 Merzhvynska M et al. *JAMA Psychiatry.*

103. Wright JH, Wright AS, Albano AM, et al. Computer-assisted cognitive therapy for depression: maintaining efficacy while reducing therapist time. *Am J Psychiatry.* 2005;162(6):1158–1164. doi:10.1176/appi.ajp.162.6.1158.

104. Yeung A, Wang F, Feng F, et al. Outcomes of an online computerized cognitive behavioral treatment program for treating chinese patients with depression: A pilot study. *Asian J Psychiatr.* 2018;38:102–107. doi:10.1016/j.ajp.2017.11.007.

105. Zu S, Xiang Y-T, Liu J, et al. A comparison of cognitive-behavioral therapy, antidepressants, their combination and standard treatment for Chinese patients with moderate-severe major depressive disorders. *J Affect Disord.* 2014;152-154:262–267. doi:10.1016/j.jad.2013.09.022.

© 2023 Merzhvynska M et al. *JAMA Psychiatry.*

eReferences 2. Literature informing the development of a Prognostic Risk Index in depression

1. Agosti V, Levin FR. The effects of alcohol and drug dependence on the course of depression. *Am J Addict.* 2006;15(1):71–75. doi:10.1080/10550490500419102.

2. Beutler LE, Castonguay LG, Follette WC. Therapeutic factors in dysphoric disorders. *J Clin Psychol.* 2006;62(6):639–647. doi:10.1002/jclp.20260.

3. Bower P, Kontopantelis E, Sutton A, et al. Influence of initial severity of depression on effectiveness of low intensity interventions: meta-analysis of individual patient data. *BMJ.* 2013;346:f540. doi:10.1136/bmj.f540.

4. Buckman JEJ, Cohen ZD, O'Driscoll C, et al. Predicting prognosis for adults with depression using individual symptom data: a comparison of modelling approaches. *Psychol Med.* 2023;53(2):408–418. doi:10.1017/S0033291721001616.

5. Buckman JEJ, Saunders R, Stott J, et al. Role of age, gender and marital status in prognosis for adults with depression: An individual patient data meta-analysis. *Epidemiol Psychiatr Sci.* 2021;30:e42. doi:10.1017/S2045796021000342.

6. Buckman JEJ, Saunders R, Cohen ZD, et al. The contribution of depressive 'disorder characteristics' to determinations of prognosis for adults with depression: an individual patient data meta-analysis. Psychol Med. 2021;51(7):1068–1081. doi:10.1017/S0033291721001367.

7. Buckman JEJ, Saunders R, O'Driscoll C, et al. Is social support pre-treatment associated with prognosis for adults with depression in primary care? *Acta Psychiatr Scand.* 2021;143(5):392–405. doi:10.1111/acps.13285.

8. Buckman JEJ, Saunders R, Stott J, et al. Socioeconomic Indicators of Treatment Prognosis for Adults With Depression: A Systematic Review and Individual Patient Data Meta-analysis. *JAMA Psychiatry.* 2022;79(5):406–416. doi:10.1001/jamapsychiatry.2022.0100.

9. Bundesärztekammer (BÄK), Kassenärztliche Bundesvereinigung (KBV), Arbeitsgemeinschaft der Wissenschaftlichen Medizinischen Fachgesellschaften (AWMF). Nationale VersorgungsLeitlinie Unipolare Depression – Langfassung, Version 3.1.

10. Cohen ZD, DeRubeis RJ. Treatment Selection in Depression. *Annu Rev Clin Psychol.* 2018;14:209–236. doi:10.1146/annurev-clinpsy-050817-084746.

11. Delgadillo J, Appleby S, Booth S, et al. The Leeds Risk Index: Field-Test of a Stratified Psychological Treatment Selection Algorithm. *Psychother Psychosom.* 2020;89(3):189–190. doi:10.1159/000505193.

12. Delgadillo J, Moreea O, Lutz W. Different people respond differently to therapy: A demonstration using patient profiling and risk stratification. *Behav Res Ther.* 2016;79:15–22. doi:10.1016/j.brat.2016.02.003.

13. DeRubeis RJ, Cohen ZD, Forand NR, Fournier JC, Gelfand LA, Lorenzo-Luaces L. The Personalized Advantage Index: translating research on prediction into individualized treatment recommendations. A demonstration. *PLoS One.* 2014;9(1):e83875. doi:10.1371/journal.pone.0083875.

14. Eaton WW, Shao H, Nestadt G, Lee HB, Bienvenu OJ, Zandi P. Population-based study of first onset and chronicity in major depressive disorder. *Arch Gen Psychiatry.* 2008;65(5):513–520. doi:10.1001/archpsyc.65.5.513.

15. Ezquiaga E, García A, Pallarés T, Bravo MF. Psychosocial predictors of outcome in major depression: a prospective 12-month study. *J Affect Disord.* 1999;52(1-3):209–216. doi:10.1016/s0165-0327(98)00057-3.

© 2023 Merzhvynska M et al. *JAMA Psychiatry.*

16. Falconnier L. Socioeconomic status in the treatment of depression. *Am J Orthopsychiatry.* 2009;79(2):148–158. doi:10.1037/a0015469.

17. Fournier JC, DeRubeis RJ, Shelton RC, Hollon SD, Amsterdam JD, Gallop R. Prediction of response to medication and cognitive therapy in the treatment of moderate to severe depression. *J Consult Clin Psychol.* 2009;77(4):775–787. doi:10.1037/a0015401.

18. Furler J, Magin P, Pirotta M, van Driel M. Participant demographics reported in "Table 1" of randomised controlled trials: a case of "inverse evidence"? *Int J Equity Health.* 2012;11:14. doi:10.1186/1475-9276-11-14.

19. Garfield SL, Bergin AE. *Bergin and Garfield's handbook of psychotherapy and behavior change.* 50th ed. Hoboken, NJ: John Wiley & Sons; 2021.

20. Hasin DS, Tsai WY, Endicott J, Mueller TI, Coryell W, Keller M. Five-year course of major depression: effects of comorbid alcoholism. *J Affect Disord.* 1996;41(1):63–70. doi:10.1016/0165-0327(96)00068-7.

21. Karsten J, Hartman CA, Smit JH, et al. Psychiatric history and subthreshold symptoms as predictors of the occurrence of depressive or anxiety disorder within 2 years. *Br J Psychiatry.* 2011;198(3):206–212. doi:10.1192/bjp.bp.110.080572.

22. Kessler RC, van Loo HM, Wardenaar KJ, et al. Using patient self-reports to study heterogeneity of treatment effects in major depressive disorder. *Epidemiol Psychiatr Sci.* 2017;26(1):22–36. doi:10.1017/S2045796016000020.

23. Kessler RC. The potential of predictive analytics to provide clinical decision support in depression treatment planning. *Curr Opin Psychiatry.* 2018;31(1):32–39. doi:10.1097/YCO.0000000000000377.

24. Moriarty AS, Meader N, Snell KIE, et al. Predicting relapse or recurrence of depression: systematic review of prognostic models. *Br J Psychiatry.* 2022;221(2):448–458. doi:10.1192/bjp.2021.218.

25. National Institute for Health and Care Excellence (NICE). Depression in adults: treatment and management. 2022:1–113.

26. Newton-Howes G, Tyrer P, Johnson T, et al. Influence of personality on the outcome of treatment in depression: systematic review and meta-analysis. *J Pers Disord.* 2014;28(4):577–593. doi:10.1521/pedi_2013_27_070.

27. O'Neill J, Tabish H, Welch V, et al. Applying an equity lens to interventions: using PROGRESS ensures consideration of socially stratifying factors to illuminate inequities in health. *J Clin Epidemiol.* 2014;67(1):56–64. doi:10.1016/j.jclinepi.2013.08.005.

28. Reich J. The effect of Axis II disorders on the outcome of treatment of anxiety and unipolar depressive disorders: a review. *J Pers Disord.* 2003;17(5):387–405. doi:10.1521/pedi.17.5.387.22972.

29. Szádóczky E, Rózsa S, Zámbori J, Füredi J. Predictors for 2-year outcome of major depressive episode. *J Affect Disord.* 2004;83(1):49–57. doi:10.1016/j.jad.2004.05.001.

30. Thase ME, Greenhouse JB, Frank E, et al. Treatment of major depression with psychotherapy or psychotherapy-pharmacotherapy combinations. *Arch Gen Psychiatry.* 1997;54(11):1009–1015. doi:10.1001/archpsyc.1997.01830230043006.

31. van Beljouw IM, Verhaak PF, Cuijpers P, van Marwijk HW, Penninx BW. The course of untreated anxiety and depression, and determinants of poor one-year outcome: a one-year cohort study. *BMC Psychiatry.* 2010;10:86. doi:10.1186/1471-244X-10-86.

© 2023 Merzhvynska M et al. *JAMA Psychiatry.*

32. World Health Organization. *Closing the gap in a generation: Health equity through action on the social determinants of health Commission on Social Determinants of Health final report.* Geneva Switzerland: World Health Organization Commission on Social Determinants of Health; 2008.

© 2023 Merzhvynska M et al. *JAMA Psychiatry.*
