# Supplementary material for: Prognostic Risk Factors in Randomized Clinical Trials of Face-to-Face and Internet-Based Psychotherapy for Depression: A Systematic Review and Meta-Regression Analysis
Source: JAMA Psychiatry. 2023 Oct 11;81(1):97–100. doi: 10.1001/jamapsychiatry.2023.3861 (PMC10568439; doi:10.1001/jamapsychiatry.2023.3861)
Supplement: Supplement 2. — Data Sharing Statement [file jamapsychiatry-e233861-s002.pdf]

Data Sharing Statement

Data
Data available: Yes
Data types: Deidentified participant data, Data (not involving human participants), Data dictionary
How to access data: https://osf.io/yspr6
When available: With publication

Supporting Documents
Document types: Other (please specify)
Additional Information: Codebook
How to access documents: https://osf.io/yspr6
When available: With publication

Additional Information
Who can access the data: Anyone requesting the data
Types of analyses: For any purpose
Mechanisms of data availability: With investigator support
